# Exploring the Potential Molecular Mechanisms of Interactions between a Probiotic Consortium and Its Coral Host

**DOI:** 10.1128/msystems.00921-22

**Published:** 2023-01-23

**Authors:** Phillipe M. Rosado, Pedro M. Cardoso, João G. Rosado, Júnia Schultz, Ulisses Nunes da Rocha, Tina Keller-Costa, Raquel S. Peixoto

**Affiliations:** a Red Sea Research Centre, King Abdullah University of Science and Technology, Thuwal, Saudi Arabia; b Biological and Environmental Science and Engineering Division, King Abdullah University of Science and Technology, Thuwal, Saudi Arabia; c Department of Environmental Microbiology, Helmholtz Centre for Environmental Research-UFZ, Leipzig, Germany; d Institute for Bioengineering and Biosciences, Instituto Superior Técnico, Lisbon, Portugal; e Institute for Health and Bioeconomy, Instituto Superior Técnico, Lisbon, Portugal; University of Pretoria

**Keywords:** beneficial microorganisms for corals (BMCs), coral probiotics, genomes, mechanisms, symbiosis, molecular interactions, holobiont, BMCs, probiotics

## Abstract

Beneficial microorganisms for corals (BMCs) have been demonstrated to be effective probiotics to alleviate bleaching and mitigate coral mortality *in vivo*. The selection of putative BMCs is traditionally performed manually, using an array of biochemical and molecular tests for putative BMC traits. We present a comprehensive genetic survey of BMC traits using a genome-based framework for the identification of alternative mechanisms that can be used for future *in silico* selection of BMC strains. We identify exclusive BMC traits associated with specific strains and propose new BMC mechanisms, such as the synthesis of glycine betaine and ectoines. Our roadmap facilitates the selection of BMC strains while increasing the array of genetic targets that can be included in the selection of putative BMC strains to be tested as coral probiotics.

**IMPORTANCE** Probiotics are currently the main hope as a potential medicine for corals, organisms that are considered the marine “canaries of the coal mine” and that are threatened with extinction. Our experiments have proved the concept that probiotics mitigate coral bleaching and can also prevent coral mortality. Here, we present a comprehensive genetic survey of probiotic traits using a genome-based framework. The main outcomes are a roadmap that facilitates the selection of coral probiotic strains while increasing the array of mechanisms that can be included in the selection of coral probiotics.

## INTRODUCTION

Changes in microbial diversity and activity affect the resilience of their hosts and therefore their ability to respond to climate change (1). Corals are model examples because associated microorganisms play a critical role in their development and growth (2, 3), in the control of pathogens (4–7), and in biogeochemical cycles (8) such as nitrogen, carbon, and sulfur cycling (9, 10), also providing access to different types of nutrients (11).

It is well known that climate change can destabilize host-associated microbiomes, leading to a state of dysbiosis, which is a disruption of the symbiotic relationships between the host and its associated microorganisms (12, 13). However, this cause-and-effect relationship is still poorly explored. Some coral species are tolerant to environmental changes and are able to maintain a relatively stable microbiome even at low pH (14) or elevated temperatures (15, 16), while other species go through a microbiome restructuring in response to impacts, such as increased seawater temperature (17, 18) and exposure to wastewater (19) and oil spills (20, 21). Some coral microbiomes can return to their original microbiome state after the stressor is removed (22, 23), while others change irreversibly to a new microbiome state that can be either beneficial or harmful to the holobiont (24).

The extensive taxonomic and metabolic diversity of the microbiome, as well as its plasticity and notably shorter generation times (compared to the coral host), provides a possible role for microorganisms in the adaptive response of the holobiont (25–30). Advantageous modifications to the microbiome could potentially be transferred vertically to subsequent generations of corals, thereby increasing the resistance of future generations to environmental stresses (31–34).

A growing appreciation of the role that microorganisms play in maintaining animal health and ecosystem functioning (27), along with the realization of possible implications for the conservation and management of endangered species (28, 35), has led some researchers to explore whether microbiome manipulation could be used to help protect and restore coral reefs (3, 6, 7, 10, 20, 21, 26, 36–39). One approach is the use of specific probiotics for corals (40), the so-called beneficial microorganisms for corals (BMCs) (26). Different members of the coral microbiome may perform the same beneficial functions (i.e., functional redundancy) (10). Therefore, one of the aims of using BMCs is to increase (or retain, as some beneficial microbes may be replaced by pathogens, during thermal stress events) the number of common, abundant, and native microorganisms that can contribute certain functions in corals undergoing stress (10, 26, 27).

Rosado et al. (7) tested the application of a BMC consortium composed of seven bacteria, including five belonging to the Pseudoalteromonas genus, one to the Cobetia genus, and one to the Halomonas genus (Table S1). All of them were isolated from the coral Pocillopora damicornis and used in a mesocosm experiment, mimicking the increase in temperature and the addition of a known coral pathogen (Vibrio coralliilyticus strain ATCC BAA-450). The experiment was conducted at two different temperatures (26°C and 30°C), comparing four treatments (BMC, BMC with a pathogen, pathogen, and control). The results of their study showed that a BMC consortium could indeed minimize the effects of both coral bleaching and pathogen challenge. Metrics used to calculate bleaching severity were significantly reduced in corals inoculated with a BMC consortium in contrast to the control treatments or treatments with pathogen addition without BMC consortia, which exhibited strong bleaching signals. For instance, the *Fv*/*Fm* ratios, a metric that assesses the Symbiodiniaceae photosystem function, were higher in treatments with inoculation of the probiotic consortium compared to the control and the treatment with pathogen inoculation.

10.1128/msystems.00921-22.2 TABLE S1 A summary of the BMC inoculation experiment performed by Rosado et al. (7). The screening was performed based on putative BMC traits proposed in the literature (26), antagonistic activity against the pathogen V. coralliilyticus, catalase production, presence of genes related to nitrogen cycling (*nirK* and *nifH*), and dimethylsulfoniopropionate (DMSP) degradation (*dmdA*). Download Table S1, DOCX file, 0.01 MB. Copyright © 2023 Rosado et al. 2023 Rosado et al. https://creativecommons.org/licenses/by/4.0/ This content is distributed under the terms of the Creative Commons Attribution 4.0 International license.

The purpose of this work is to perform an *in silico* analysis of the genomes of the P. damicornis BMCs used by Rosado et al. (7), aiming to identify potential molecular mechanisms of interaction between members of the consortium and the host that may in turn guide further selections of novel BMCs. In this regard, our *in silico* analysis can support the development of a framework for the selection of customized consortia with specific BMC characteristics for specific hosts and stress conditions, which can accelerate and optimize the selection of BMC consortia. However, it is important to emphasize that the beneficial characteristics for corals analyzed in this work are mainly theoretical and based on the literature and therefore require validation by, for example, combined physiological and molecular (such as metatranscriptomics and other omics) monitoring during laboratory experiments and field trials.

## RESULTS

### Genomic features of the BMC strains.

A combined total of 11,789,856 clean and trimmed high-quality reads were obtained from Illumina sequence reads. The genome assembly of the seven BMC strains performed using SPAdes resulted in a total of 68 to 222 contigs/strain (total contigs obtained). CheckM analysis revealed a high genome completeness (>99% and 100% complete) and a low level of contamination (<1%) for all genomes, except BMC 1, which, after using RefineM to improve the quality, reached a completeness of 87.58% while contamination dropped from 31.25% to 0.19%. General features from the draft genomes of the BMC strains are summarized in Table S4.

10.1128/msystems.00921-22.5 TABLE S4 General genome characteristics of the seven BMC strains. Download Table S4, DOCX file, 0.01 MB. Copyright © 2023 Rosado et al. 2023 Rosado et al. https://creativecommons.org/licenses/by/4.0/ This content is distributed under the terms of the Creative Commons Attribution 4.0 International license.

The Pseudoalteromonas sp. genomes of BMC strains 1 to 5 presented very similar characteristics, with a G + C content of 41% and an average size of 4.5 Mbp. Notably, the values of the number of contigs, tRNAs, rRNAs, and coding genes were lower in BMC 1 compared to BMCs 2, 3, 4, and 5, probably due to the refinement of this genome, which resulted in a loss of 12% of its gene content. The genomes of BMC strains 6 and 7, previously identified as different genera (Cobetia and Halomonas, respectively), showed very similar characteristics to each other, such as a G + C content of 62.47% and 61.60%, respectively, and an average genome size of 3.9 Mbp.

### Taxonomic attribution.

To identify each BMC strain, two different genome alignment methods (FastANI and Genome-to-Genome Distance Calculator [GGDC]) were used to mimic conventional DNA-DNA hybridization (DDH). Pairwise comparison between BMCs 1 to 5 using the GGDC index obtained a similarity value of 100%, while the average nucleotide identity (ANI) indicated a similarity between 97.28% and 99.99%, confirming that they belong to the same species (Table S2).

10.1128/msystems.00921-22.3 TABLE S2 Taxonomic assignment of all BMC genomes using two different tools. The genome-to-genome distance calculator (GGDC) was used to calculate DNA-DNA hybridization values, and FastANI was used to calculate ANI values. Download Table S2, DOCX file, 0.01 MB. Copyright © 2023 Rosado et al. 2023 Rosado et al. https://creativecommons.org/licenses/by/4.0/ This content is distributed under the terms of the Creative Commons Attribution 4.0 International license.

Four publicly available Pseudoalteromonas genomes obtained a DDH estimate higher than 70% (Pseudoalteromonas sp. CO109Y, Pseudoalteromonas shioyasakiensis JCM 18891, P. shioyasakiensis M1400201, and Pseudoalteromonas sp. P1-8), and five obtained an ANI value higher than 95% (the previous four Pseudoalteromonas plus P. shioyasakiensis SDCH90) when aligned with all Pseudoalteromonas BMCs (Table S2). The cutoff values used for organisms to be considered the same species were 70% and 95% for the DDH estimate and ANI value, respectively. Based on these results, it is possible to conclude that BMCs 1 to 5 belong to the same species, P. shioyasakiensis.

**FIG 1:**
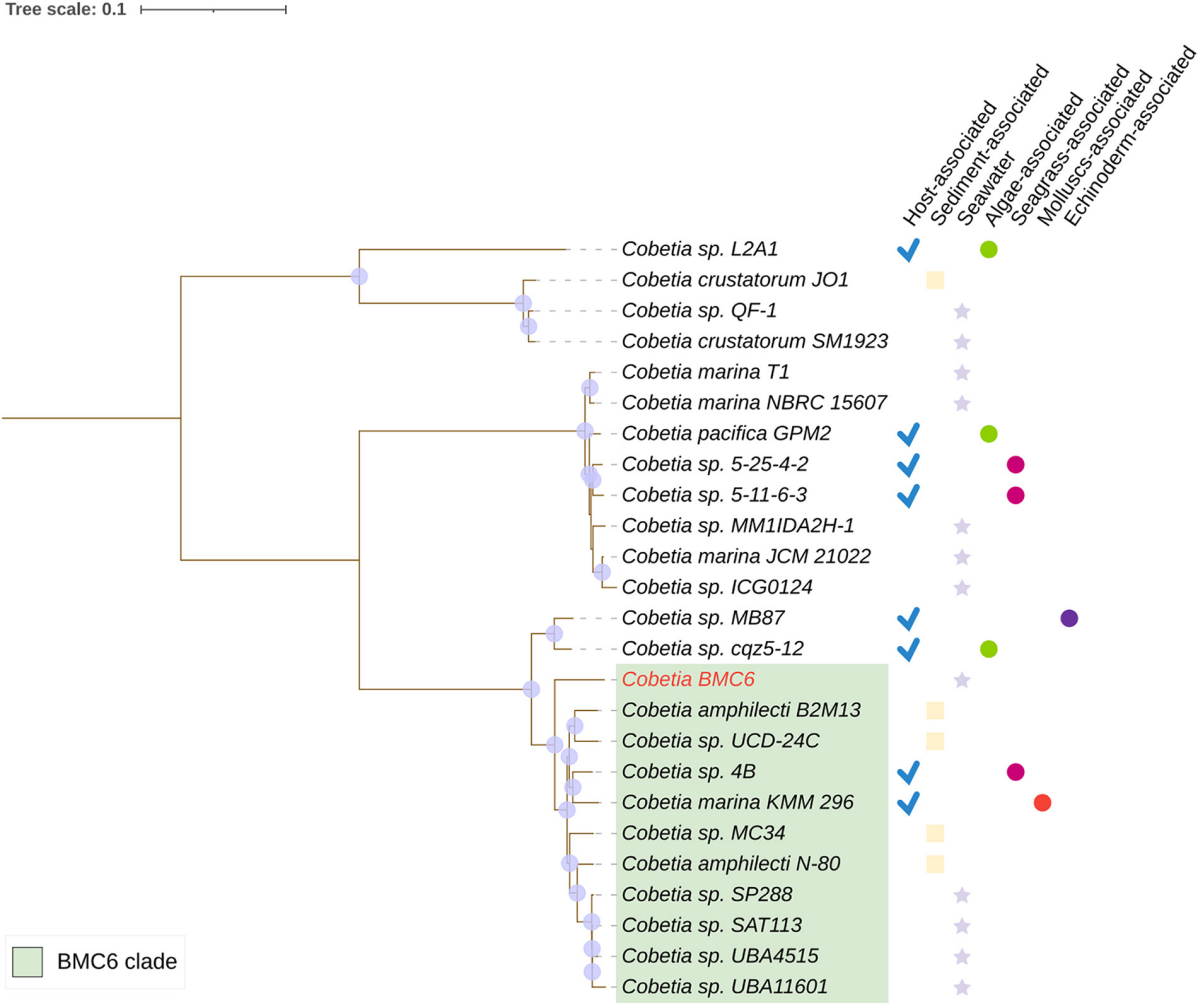
Phylogenomic inference of publicly available genomes from 24 Cobetia strains and the genomes of beneficial microorganisms for coral strain 6 (BMC6) (in red), totaling 25 genomes. The green-shaded area represents the strains that form a monophyletic clade with BMC6. The tree was assembled from the comparison of 1,000 proteins through the codon tree method of the PATRIC platform that selects global protein families (PGFams) as homology groups and analyzes aligned proteins and DNA encoding single-copy genes using the RAxML program. The best protein model found by RAxML to build this tree was Jones-Taylor-Thornton (JTT). Purple dots on the branch length correspond to the bootstrap value of 100. The icons on the right side of the strains represent their isolation sources.

**FIG 2:**
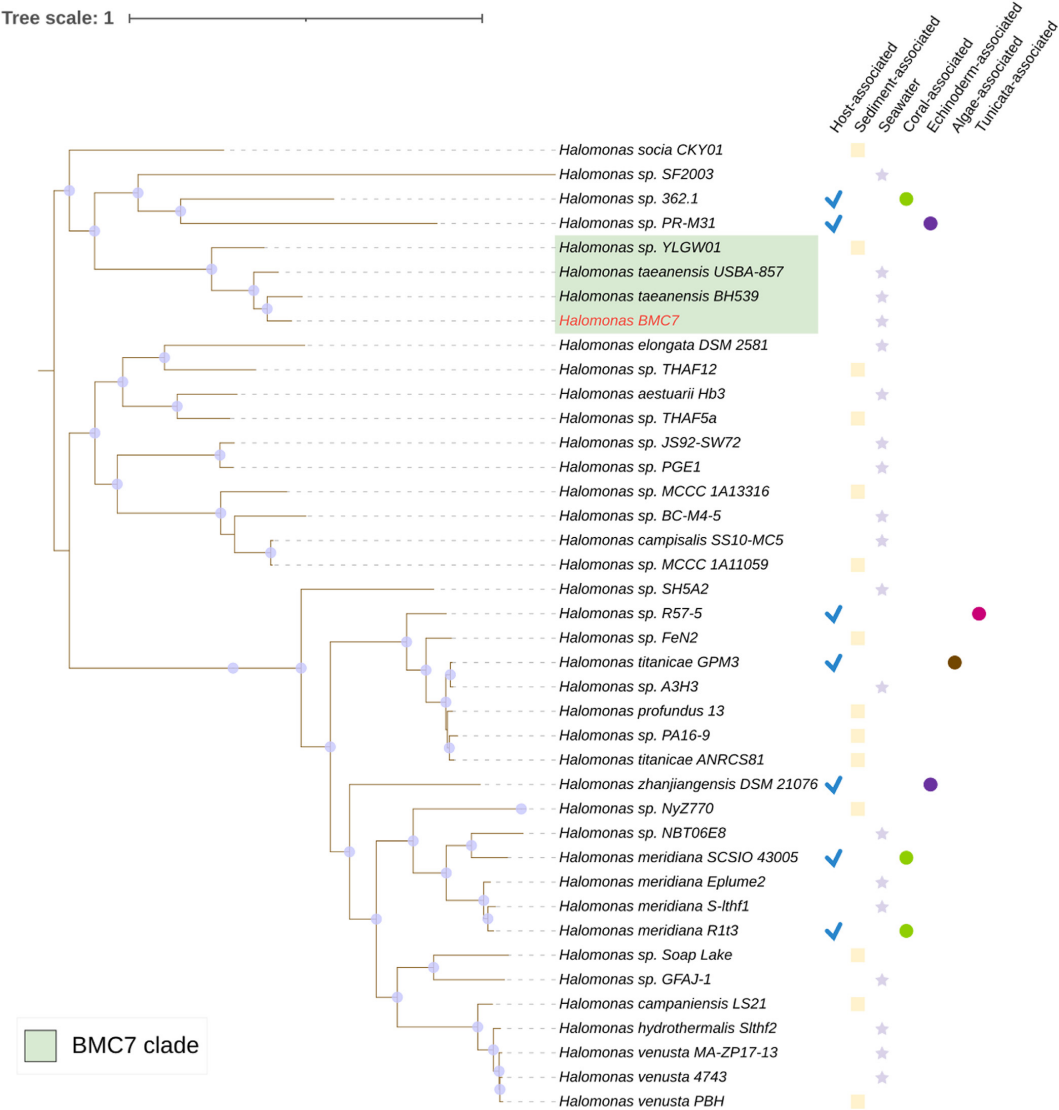
Phylogenomic inference of publicly available genomes from 39 Halomonas strains and the genomes of BMC7 strain (in red), totaling 40 genomes. The green-shaded area represents the strains that form a monophyletic clade with BMC7. The tree was assembled from the comparison of 1,000 proteins through the codon tree method of the PATRIC platform that selects global protein families (PGFams) as homology groups and analyzes aligned proteins and DNA encoding single-copy genes using the RAxML program. The best protein model found by RAxML to build this tree was LG. Purple dots on the branch length correspond to the bootstrap value of 100. The icons on the right side of the strains represent their isolation sources.

The same analyses were performed for BMCs 6 and 7 (Table S2), previously identified (16S rRNA gene analysis) as Cobetia marina and Halomonas taeanensis, respectively (7). Ten genomes belonging to the genus Cobetia were used in the comparison with the genome of BMC 6 (Fig. 1; Table S2). The ANI taxonomic assignment method indicated that all 10 genomes shared more than 95% similarity. However, the DDH estimate indicated that none of the 10 genomes showed more than 70% similarity. The similarity values obtained from the comparison between BMC 7 and publicly available reference/closest Halomonas genomes were below the cutoff value for species identification for both ANI and GGDC methods (i.e., 91.31% and 41.6%, respectively) (Fig. 2; Table S2).

### Phylogenomic tree assembly using the draft genomes of BMCs.

**FIG 3:**
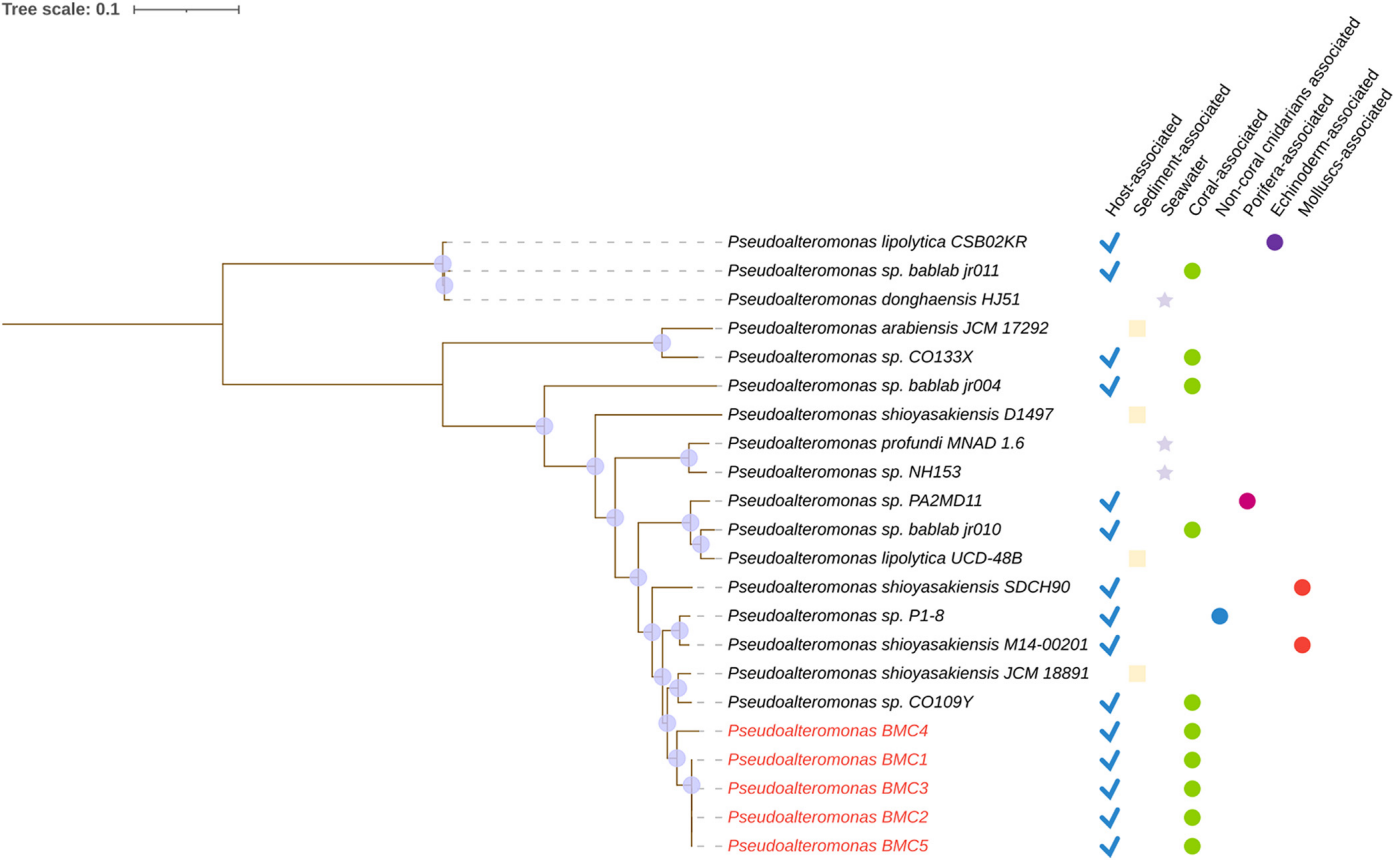
Phylogenomic inference of publicly available genomes from 17 Pseudoalteromonas strains and the genomes of BMC strains 1 to 5 (in red), totaling 22 genomes. The tree was assembled from the comparison of 1,000 proteins through the codon tree method of the PATRIC platform that selects global protein families (PGFams) as homology groups and analyzes aligned proteins and DNA encoding single-copy genes using the RAxML program. The best protein model found by RAxML to build this tree was Jones-Taylor-Thornton - Direct Computation with Mutabilities (JTTDCMut). Purple dots on the branch length correspond to the bootstrap value of 100. The icons on the right side of the strains represent their isolation sources.

The phylogenomic relationships between the seven BMC genomes and the closest publicly available genomes were evaluated. In the assembly of the phylogenomic tree of Pseudoalteromonas sp. BMCs, 90 genomes belonging to the genus Pseudoalteromonas were used (Fig. S1). In this phylogenomic tree, a well supported clade, formed by the BMCs 1, 2, 3, 4, and 5 and 17 other Pseudoalteromonas sp. strains, was identified. Subsequently, another tree was assembled using only the genomes of this P. shioyasakiensis BMC clade (Fig. 3). Of the 17 strains used in this phylogenomic tree, 10 were isolated from a host, while 7 were isolated from either sediment or seawater. Among the hosts were corals, cnidarians, mollusks, sponges, and echinoderms.

10.1128/msystems.00921-22.1 FIG S1 Phylogenomic inference of publicly available genomes from 90 Pseudoalteromonas strains and the genomes of BMC strains 1 to 5 (in red), totaling 95 genomes. The tree was assembled from the comparison of 100 proteins through the codon tree method of the PATRIC platform that selects global protein families (PGFams) as homology groups and analyzes aligned proteins and DNA encoding single-copy genes using the RAxML program. The best protein model found by RAxML to build this tree was LG. Purple dots on the branches correspond to the bootstrap value of 100. Download FIG S1, TIF file, 1.1 MB. Copyright © 2023 Rosado et al. 2023 Rosado et al. https://creativecommons.org/licenses/by/4.0/ This content is distributed under the terms of the Creative Commons Attribution 4.0 International license.

For the phylogenomic inference of Cobetia sp. BMC 6, another 24 genomes of strains belonging to the genus Cobetia were used. Cobetia sp. BMC 6 formed a clade with 10 other Cobetia sp. genomes. Of these 10 strains, only 2 were isolated from a host (seagrass and mollusk), while 4 were isolated from sediment, and 4 were isolated from seawater (Fig. 1).

In the Halomonas sp. BMC tree, 39 other publicly available genomes were included. The formation of a small clade between Halomonas sp. BMC 7 and three other strains of Halomonas sp. (YLGW01, H. taeanensis USBA-857, and H. taeanensis BH539) was observed (Fig. 2). None of the three strains were isolated from a host; one was isolated from sediment, and the other two, USBA-857 and BH539, were from seawater.

### Gene functions with potential benefits for corals.

Genes encoding proteins that may be related to potentially beneficial characteristics for corals (26, 41) or for their endosymbiont algae (42) were selected using PATRIC. Subsystems related to overall potentially beneficial traits described by Peixoto et al. (26, 41) and Matthews et al. (42) were screened; genes linked to oxidative stress, nitrogen cycling, cobalamin (vitamin B_12_) synthesis, siderophore production (i.e., production of iron-chelating compounds), dimethylsulfoniopropionate (DMSP) degradation, glycine betaine production, and ectoine synthesis were identified (Table S5).

10.1128/msystems.00921-22.6 TABLE S5 Some examples of gene functions related to putative beneficial traits for coral found in the BMC genomes. Download Table S5, DOCX file, 0.01 MB. Copyright © 2023 Rosado et al. 2023 Rosado et al. https://creativecommons.org/licenses/by/4.0/ This content is distributed under the terms of the Creative Commons Attribution 4.0 International license.

The subsystems of interest found in the genomes of P. shioyasakiensis BMCs 1 to 5 included oxidative stress protection, cobalamin synthesis, and glycine betaine synthesis. The number of annotated genes belonging to these subsystems was similar among all BMC bacteria of the species. Cobetia sp. BMC 6 and Halomonas sp. BMC 7 shared subsystems related to oxidative stress, nitrogen cycling, siderophore synthesis, glycine betaine production, ectoine synthesis, and DMSP degradation. Halomonas sp. BMC 7 also harbored genes for cobalamin synthesis, which were absent in Cobetia sp. BMC 6.

### Pangenome comparison and categorization of singleton genes.

**FIG 4:**
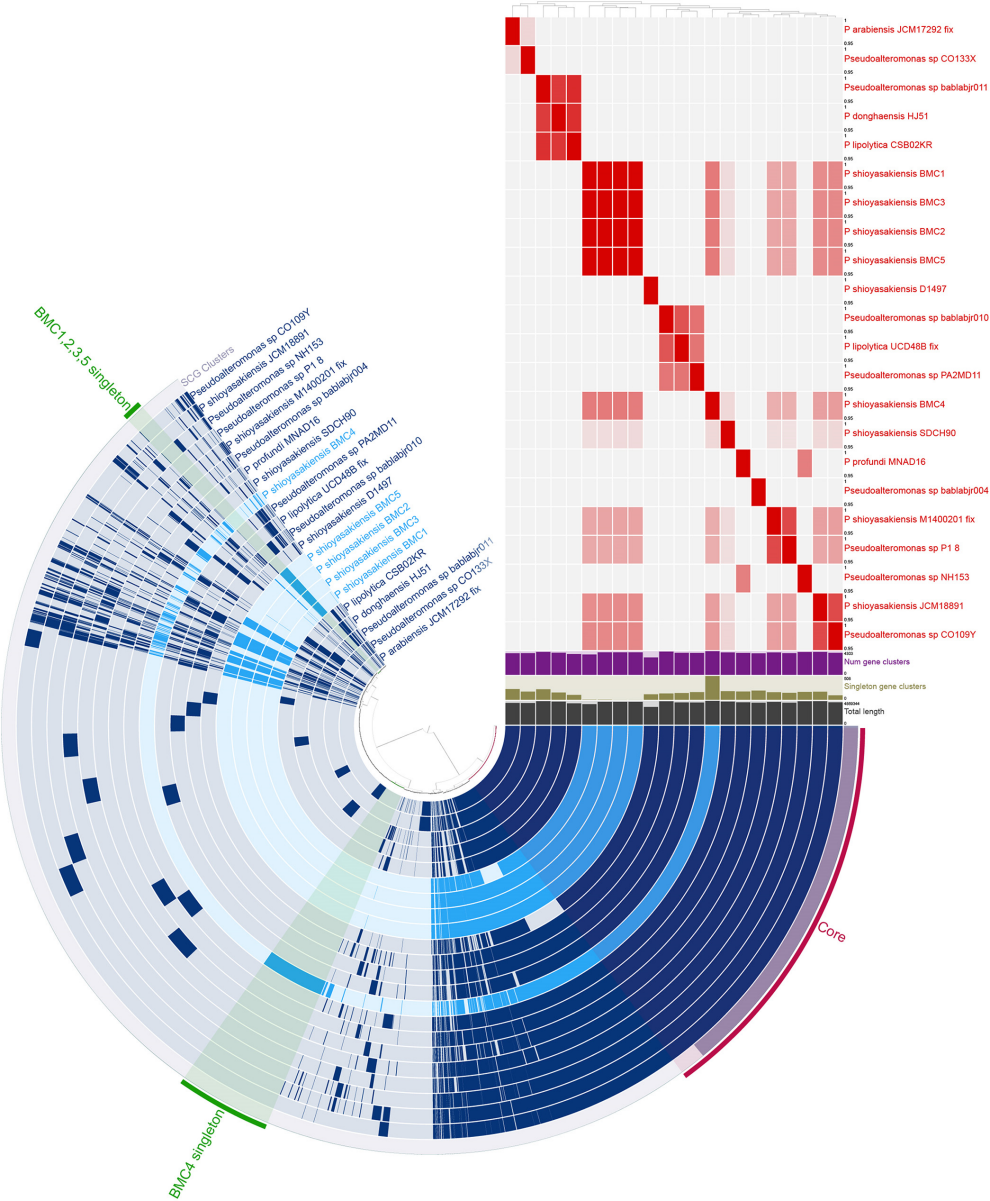
Pangenomic analysis of Pseudoalteromonas strains. The outermost circle displays the single-copy genes (SCG cluster) that were shared by all the genomes. The presence and absence of coding sequences (CDS) in the genome are indicated in dark blue and light blue, respectively. The average nucleotide identity (ANI) (95% to 100%) comparison among all 22 genomes is displayed with the red and white heat map. The dendrogram at the top of the heat map represents the hierarchical clustering of genomes based on the occurrence of gene clusters. The layers underneath the %ANI heat map, from top to bottom, indicate number of gene clusters (purple), number of singleton gene clusters (dark green), and total length (gray) of each genome.

**FIG 5:**
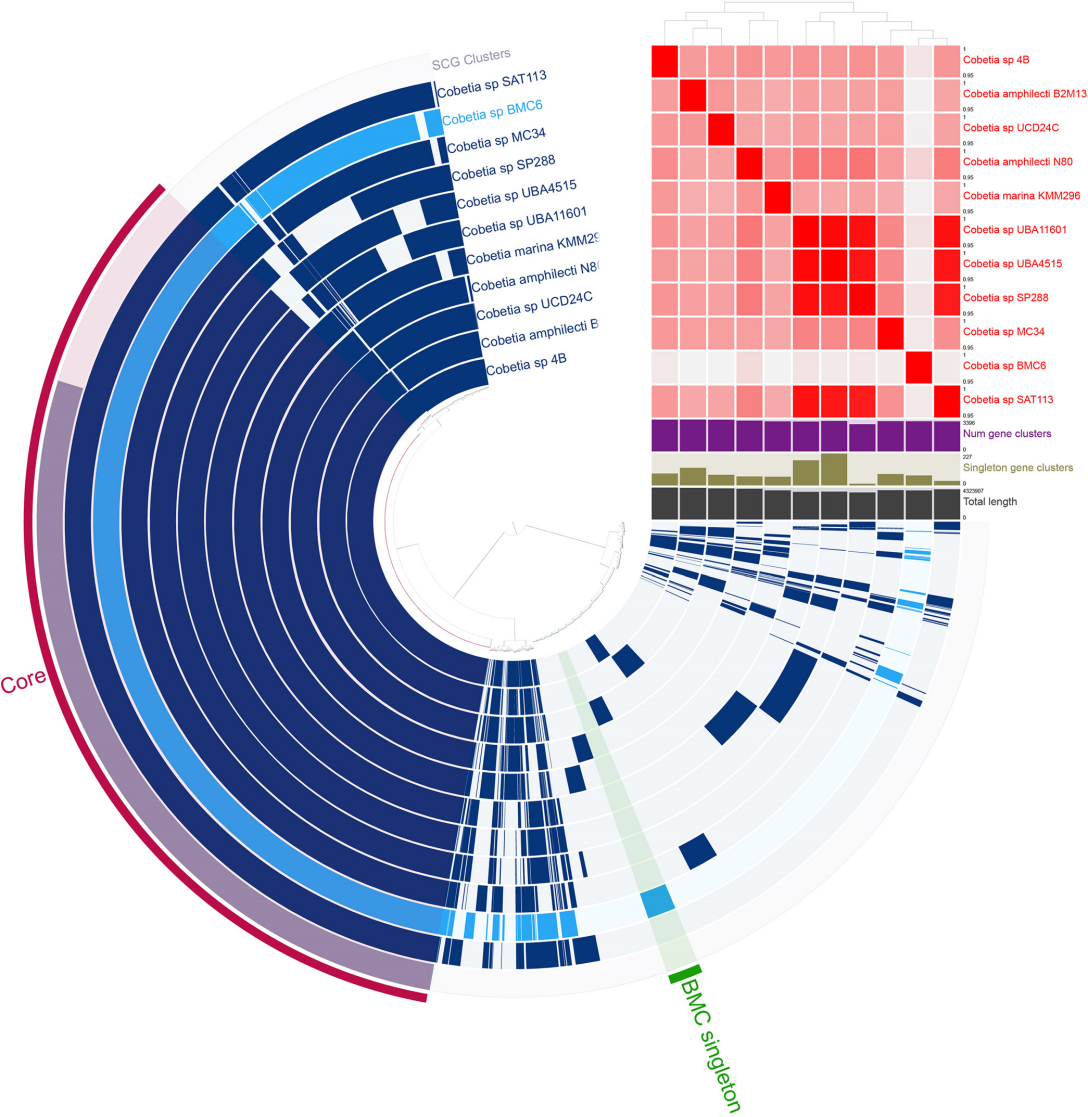
Pangenomic analysis of Cobetia strains. The outermost circle displays the single-copy genes (SCG cluster) that were shared by all the genomes. The presence and absence of coding sequences (CDS) in the genome are indicated in dark blue and light blue, respectively. The ANI (95% to 100%) comparison among all 11 genomes is displayed with the red and white heat map. The dendrogram at the top of the heat map represents the hierarchical clustering of genomes based on the occurrence of gene clusters. The layers underneath the %ANI heat map, from top to bottom, indicate number of gene clusters (purple), number of singleton gene clusters (dark green), and total length (gray) of each genome.

**FIG 6:**
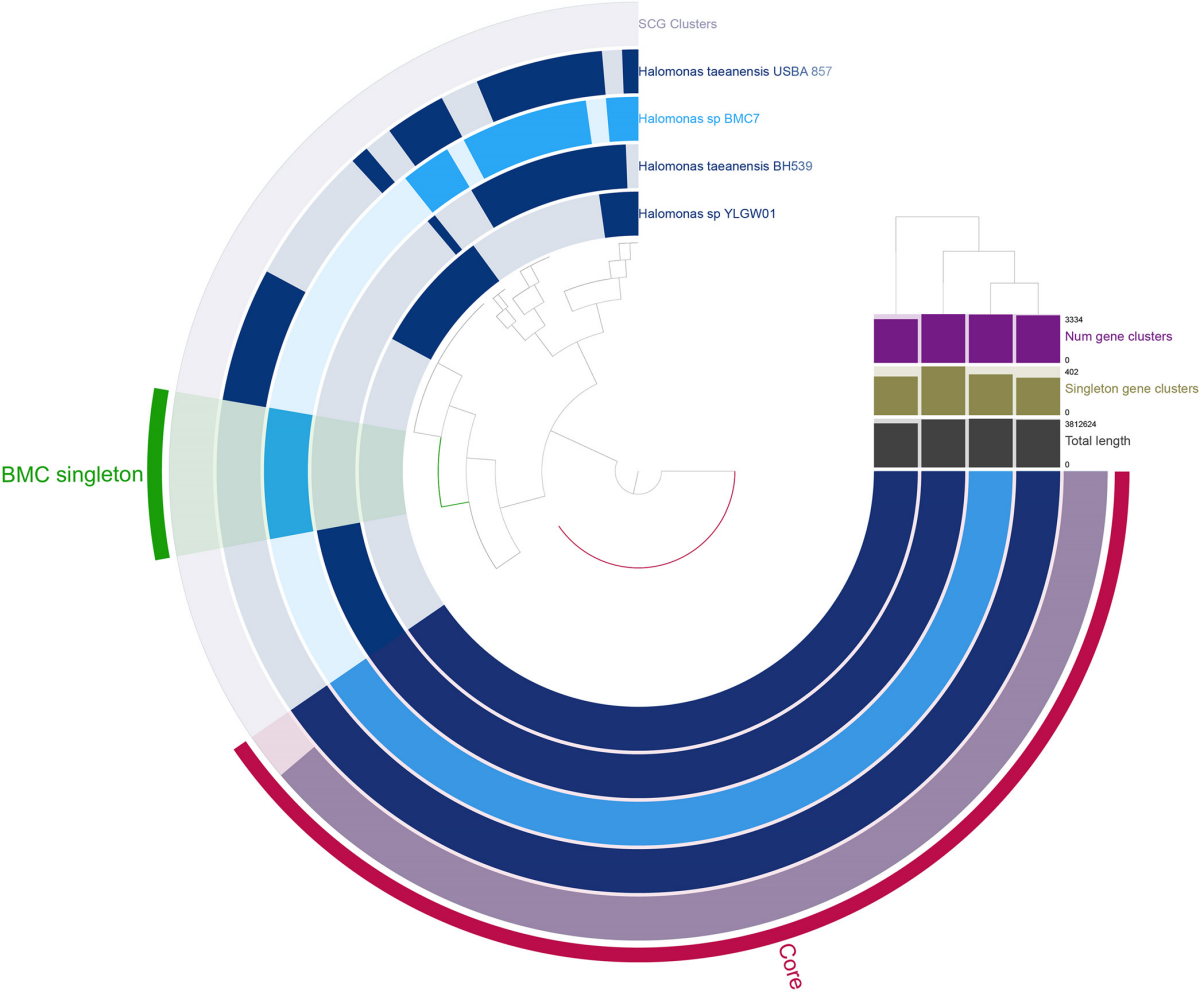
Pangenomic analysis of Halomonas strains. The outermost circle displays the single-copy genes (SCG cluster) that were shared by all the genomes. The presence and absence of coding sequences (CDS) in the genome are indicated in dark blue and light blue, respectively. The dendrogram at the top of the number of gene clusters represents the hierarchical clustering of genomes based on the occurrence of gene clusters. The layers underneath the dendrogram, from top to bottom, indicate number of gene clusters (purple), number of singleton gene clusters (dark green), and total length (gray) of each genome.

A pangenomic comparison was performed to identify genes that were unique to specific BMC strains. Pangenomes were independently generated for each BMC clade (Fig. 4 to 6). Seventeen additional Pseudoalteromonas strains were added to the pangenome of P. shioyasakiensis BMC strains 1 to 5, which formed a BMC clade (Fig. 3). A total of 1,076 unique BMC genes were identified in this pangenome (i.e., genes that were present only in the genome of one or more P. shioyasakiensis BMCs 1 to 5, but not in the genomes of the other 17 Pseudoalteromonas strains). Of these, the functions of only approximately one-third (208 genes) are known, while two-thirds (868 genes) were classified as hypothetical proteins. Among the 208 genes with known functions, 8 were shared by all P. shioyasakiensis BMC strains, 49 were present in four BMC strains, 7 genes were found only in three BMC strains, 1 gene was detected only in two BMC strains, and 143 genes were exclusive to BMC 4 (Fig. 4). Of the 208 unique BMC genes with known functions, 2 encoded previously proposed beneficial traits for corals (Table S6), both related to iron bioavailability.

10.1128/msystems.00921-22.7 TABLE S6 Proteins identified in the P. shioyasakiensis BMC 1 to 5, Cobetia sp. BMC 6, and Halomonas sp. BMC 7 clade pangenomes that are present only in the BMCs and are likely to benefit the coral. Download Table S6, DOCX file, 0.01 MB. Copyright © 2023 Rosado et al. 2023 Rosado et al. https://creativecommons.org/licenses/by/4.0/ This content is distributed under the terms of the Creative Commons Attribution 4.0 International license.

A total of 382 singleton genes were identified for Cobetia sp. BMC 6, compared to the other 10 Cobetia genomes used (Fig. 5). Of these, 162 genes were known, while 220 were classified as hypothetical proteins. Six of the known genes encoded previously defined beneficial traits for corals (Table S6) related to sulfur cycling, iron bioavailability, antibiotic production, vitamin B synthesis, nitrogen cycling, and oxidative stress protection.

**FIG 7:**
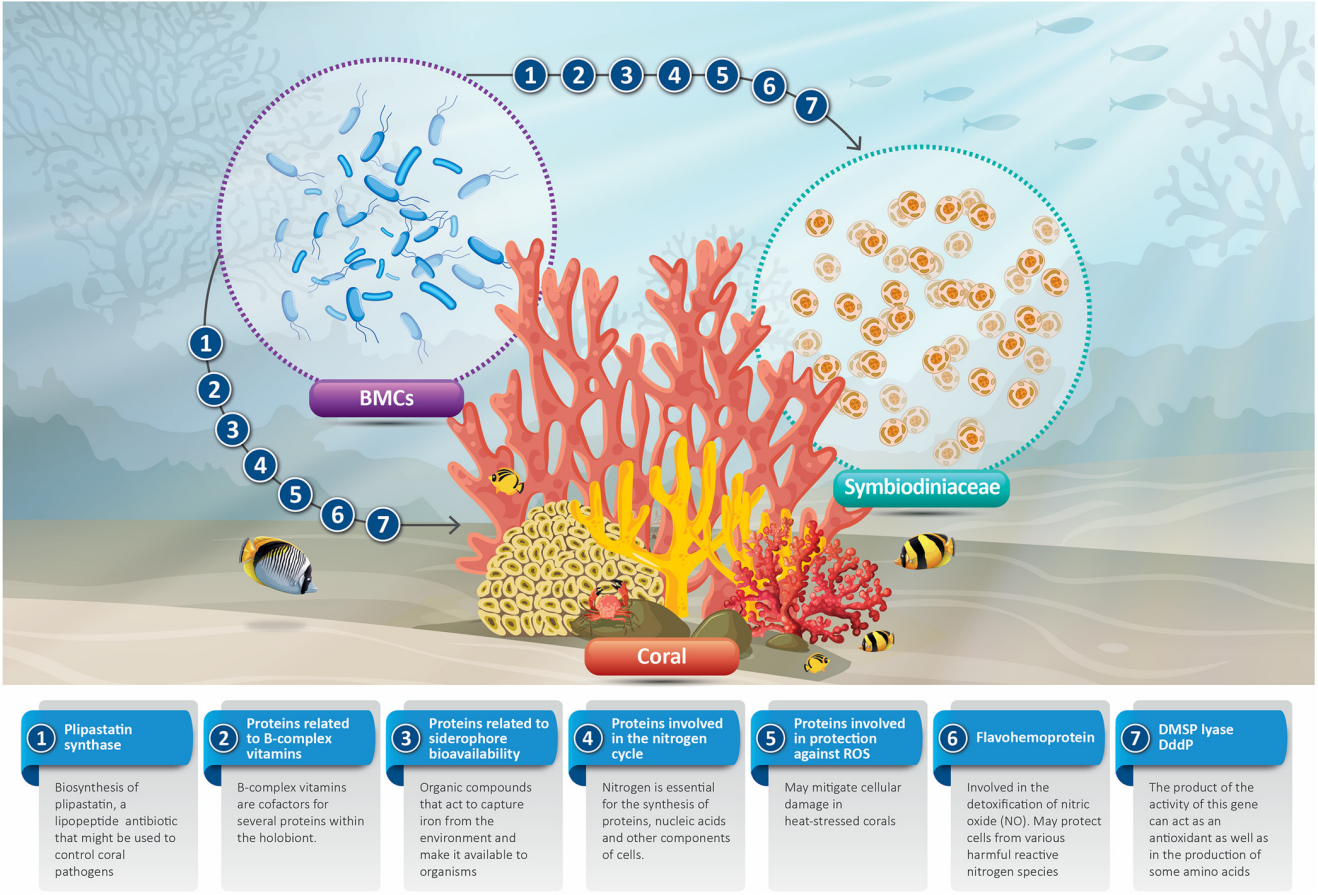
Theoretical illustration of the benefits that singleton genes of BMCs might bring to the coral host and its endosymbiotic algae.

The phylogenomic assessment indicated that Halomonas sp. BMC 7 formed a clade with the other three Halomonas sp. strains used for the pangenome analysis (Fig. 2). A total of 1,447 genes were identified as singletons, which were detected only in the genome of Halomonas sp. BMC 7 (Fig. 6). Among the 1,447 genes, 737 genes encoded hypothetical proteins, while 710 genes have a known function. Among these 710 genes with known functions, 18 genes encode previously proposed beneficial traits for corals (Table S6) related to siderophores production, vitamin B complex synthesis, sulfur cycling, response to oxidative stress, nitric oxide detoxification, and nitrogen cycling, among others. Fig. 7 summarizes the beneficial features of BMC singleton genes for coral and algal endosymbionts.

## DISCUSSION

### Cobetia sp. BMC 6 and Halomonas sp. BMC 7 are new species candidates.

ANI and DDH values, together with our phylogenomic inference, confirmed that BMC strains 1 to 5 belong to the same species, namely, P. shioyasakiensis, which was initially described and isolated from marine sediment (43). The difference in genome size and ANI of BMC 4 compared to BMCs 1, 2, 3, and 5 indicates that BMC 4 may be a more divergent strain or even a subspecies of P. shioyasakiensis.

The higher similarity between the genomes of BMC strains 6 and 7 compared to BMCs 1 to 5 was expected as the two genera, Cobetia and Halomonas, respectively, belong to the *Halomonadaceae* family and have only recently been separated into two distinct genera (44). Unlike the taxonomic and phylogenomic results obtained for the Pseudoalteromonas BMCs 1 to 5, the results of Cobetia sp. BMC 6 and Halomonas sp. BMC 7 did not allow the classification of these strains at the species level. With the ANI taxonomic attribution method, Cobetia sp. BMC 6 showed a similarity higher than 95% with the closest 10 strains (Table S2) that form a clade in the Cobetia sp. phylogenomic tree (Fig. 1). However, the GGDC index indicates the highest similarity value of 64.10% with Cobetia amphilecti N80, which is below the cutoff value of 70% defined for the classification of the same species. Considering these results together, the species definition for this BMC is inconclusive, and therefore, we suggest the use of Cobetia sp. when referring to this BMC strain. Cobetia sp. BMC 6 may represent a new species within the genus Cobetia, but further analyses, such as phenotypic and biochemical tests related to the genus and API tests, are necessary to determine this (45).

Although a phylogenomic clade was formed by Halomonas sp. BMC 7 strain with Halomonas sp. YLGW01 and H. taeanensis strains, both ANI and DDH values were below the defined species-level thresholds, and we also suggest the use of Halomonas sp. when referring to BMC 7. This BMC is possibly a new species of Halomonas, but further analyses to characterize the species are needed, as described for Cobetia sp. BMC 6. Based on this evidence, the lack of comparative genomes likely contributed to the low taxonomic resolution of both the Cobetia and the Halomonas BMCs.

### Genome analyses confirm the presence of previously suggested BMC mechanisms.

Following the phylogenomic and taxonomic inference of the BMCs, we searched their genomes for genes encoding proteins that could be potentially beneficial for corals. These genes are not exclusive to BMCs and can also be found in any non-BMC strains. In addition to genes encoding catalase and nitrite reductase enzymes identified via PCR and biochemical tests, which were used for their selection as potential BMCs (7), genes related to other putative beneficial traits were also detected. These included several genes encoding proteins with antioxidant roles, such as superoxide dismutase (present in all BMCs). Superoxide is a product of oxygen metabolism and can cause various types of cell damage (46). Another important gene associated with the antioxidant role found in all BMCs is the gene named *GSS*, which encodes the protein glutathione synthetase and is related to the glutathione biosynthesis pathway (47); glutathione can be used by the protein glutathione peroxidase, also present in all BMCs, to neutralize reactive oxygen species (ROS), such as H_2_O_2_ (48). H_2_O_2_, among other ROS, are produced in coral holobionts during periods of high temperatures and irradiance, causing damage to host and symbiont cells (49–51). The production of ROS is directly linked to coral bleaching (52, 53). ROS-scavenging BMC strains have been hypothesized to alleviate bleaching signs in stressed coral holobionts (26) and should therefore be targeted for BMC selection.

Genes associated with the synthesis of cobalamin, also known as vitamin B_12_, a cofactor involved in the production of the amino acid methionine (which is necessary to synthesize all proteins), were observed in all BMC genomes, except in Cobetia sp. BMC 6. Vitamin B_12_ biosynthesis is associated with several metabolic pathways, including the generation of glutathione and DMSP antioxidants (54), which are mechanisms that can directly help corals during heat stress by mitigating increased concentrations of ROS (26, 41), and has therefore been recently suggested as a BMC trait (41). In addition, Symbiodiniaceae is not capable of synthesizing cobalamin, so adding BMCs that may play this role would help to meet the needs of endosymbiotic algae. Again, this is theory that is described in the literature (e.g., Peixoto et al. [26, 41], Matthews et al. [42], and Santoro et al. [10]); several more specific studies are needed to substantiate this theory. Some such studies are already being performed by our laboratory, as well as in other laboratories. We believe the caveats discussed above will be important to highlight that this is a roadmap for the screening of BMCs that will be then validated using manipulative studies followed by multiomics and physiological analyses, as for example in the work of Santoro et al. (10). Algae belonging to the *Symbiodiniaceae* family require exogenous cobalamin for growth, because they do not have the genetic machinery to generate the active form of this vitamin (42), implying that *Symbiodiniaceae* rely on bacterial symbionts to access this important cofactor.

Both Cobetia sp. BMC 6 and Halomonas sp. BMC 7 also carry different genes associated with the synthesis of siderophores and ectoines (55). Siderophores are organic compounds that act to capture iron from the environment, and their production has also been suggested as a BMC trait (41). Iron is essential for several physiological processes in corals and microalgae, including photosynthesis, respiration, and nitrogen fixation (56), yet bioavailable iron concentrations in most of the global oceans are too low to support growth of microalgae (57). Several marine bacteria produce siderophores, which bind and concentrate iron in bioavailable forms, allowing the absorption of this limiting micronutrient by phytoplankton. For instance, the production of siderophores by the gammaproteobacterium Marinobacter promotes the growth of its dinoflagellate partner Scrippsiella trochoidea (58). Marinobacter strains are also a component of the “core microbiome” shared between different cultures of *Symbiodiniaceae* (59), which likely rely on bacterial partners to meet their bioavailable iron needs.

Ectoines, which include the compounds ectoine and hydroxyectoine, have several roles in the physiology of prokaryotic and eukaryotic organisms, including protection against radiation and oxidative stress, enzyme stabilization, and cryoprotection during freeze-thaw processes. Bownik et al. (60) tested the potential role of ectoines as thermal protectors in Daphnia magna, a small planktonic crustacean. Their results showed protective effects of ectoine in D. magna subjected to heat stress, which was the first indication of the beneficial effects that ectoines can generate in aquatic animals undergoing this type of stress. Therefore, we hypothesize that this type of protection can also occur in corals undergoing thermal stress, and bacteria that synthesize ectoines are potential targets for selection as BMCs. We also suggest that ectoine production may have been one of the protective mechanisms promoted by BMCs 6 and 7 in the work of Rosado et al. (7).

Furthermore, we detected genes whose functions are involved in the synthesis of glycine betaine (GB) as a common gene across our BMC strains. GB acts as a compatible solute (osmolyte) to neutralize the effects of high salinity and contributes to the protection of membranes and proteins against abiotic stressors (61, 62). Ngugi et al. (63) suggested that GB metabolism acts on coral physiology and symbiotic interactions. The authors highlighted the fact that GB comprises about 16% of the total nitrogen biomass of corals, being also a potential nitrogen source for the holobiont. In addition to its properties as an osmolyte, GB may also have additional properties that replace other osmolytes, such as DMSP. In plants, for example, GB inhibits heat and irradiance stress by stabilizing vital extrinsic proteins of the photosystem II complex (61). A similar role is hypothesized in corals and their endosymbiotic algae (64), as shallow water corals and those exposed to high irradiance have higher concentrations of GB compared to colonies that live in deeper water and shaded areas (64). Finally, GB metabolism may be a key determinant of carbon metabolism that supports methyl-dependent processes (such as DNA methylation and protein synthesis). As carbon and nitrogen metabolism are often closely linked, it is hypothesized that the symbiosis between corals and their carbohydrate-producing algal symbionts may have selectively enhanced GB sequestration and storage to support metabolic activity, maintaining a proper osmotic environment while serving as a nitrogen reservoir to accommodate the growing needs of endosymbionts (63). It is important to note that these studies did not directly test the role of GB in heat-stressed corals; thus, future research addressing this topic is required. For these reasons, in addition to the high abundance of GB-producing strains in our BMC consortium, we also suggest GB production as a BMC mechanism that was likely associated with the successful application of the consortium by Rosado et al. (7).

The BMC traits mentioned above bring direct benefits to corals and/or their endosymbiotic algae and indicate potential mechanisms that may have contributed to the bleaching mitigation observed (7), in addition to the characteristics previously used to select the BMC consortium. In some cases, they also represent new BMC targets that should be investigated in future research efforts (such as the production of different ROS scavengers, GB, and ectoines). Furthermore, transcriptomic and/or metabolomic surveys will always be required in thermal stress experiments to confirm whether these BMC mechanisms are being expressed and correlated with thermal protection (10).

### Pan-(BMC)genomes suggest additional, novel BMC mechanisms.

We also generated pangenomes for the BMCs and other non-BMC strains to identify genes that are unique to the selected BMC strains. The Pseudoalteromonas pangenome identified 208 genes with known functions that have been identified only in the BMC genomes (i.e., in one or more strains of the P. shioyasakiensis BMCs 1, 2, 3, 4, and 5) (Fig. 4). Among those genes, we identified two encoded proteins that may have a putative beneficial role, related to iron bioavailability, for corals and *Symbiodiniaceae*: ferri-bacillibactin esterase BesA (also found in Cobetia sp. BMC 6) and luminescence regulatory protein LuxO. The ferri-bacillibactin esterase hydrolyzes the ferri-bacilibactin (ferri-BB) complex during the trilactone cycle, leading to the release of cytosolic iron, making iron available for metabolic use (65). LuxO plays a role in the production of siderophores (66). As mentioned previously, iron is essential for a number of physiological processes in corals and microalgae, including photosynthesis, respiration, and nitrogen fixation (56). Notably, more than two-thirds of the genes found exclusively in P. shioyasakiensis BMCs are classified as hypothetical proteins of unknown function, and several of these genes may or may not play a vital role in the holobiont health.

The Cobetia pangenome identified 162 genes with known functions that were identified in the Cobetia sp. BMC 6 genome only (Fig. 5). Among these genes, six encode proteins that may play a putative beneficial role for corals and *Symbiodiniaceae*. These proteins include plipastatin synthase, an enzyme involved in the biosynthesis of plipastatin, an antimicrobial lipopeptide that has been reported to have a broad antagonistic effect on various soil and postharvest fungal phytopathogens, specifically on filamentous fungi (67), in addition to possessing antibacterial and antiviral activity (68–70). These genes represent specific BMC mechanisms involved in the biological control of pathogens in coral (26). The protein cyclic pyranopterin monophosphate synthase was also exclusive to the Cobetia sp. BMC 6 genome. This protein is involved in the biosynthesis of molybdopterin, an important cofactor of some enzymes that represent putative BMC mechanisms (i.e., dimethyl sulfoxide [DMSO] reductase and nitrate reductase) (71). Another protein exclusive of the Cobetia sp. BMC 6 genome is heme A synthase, a component of many biologically important hemoproteins, which include proteins related to protection against oxidative stress, such as catalases and heme peroxidase (72).

The protein siroheme synthase is encoded by the *cysG* gene and was present only in the genomes of Cobetia sp. BMC 6 and Halomonas sp. BMC 7, being absent in the other genomes used in the pangenome analyses of both genera. This may have occurred because there were limited data for these pangenome analyses, particularly for the Halomonas genus, for which there were only three representative genomes. This protein is involved in sulfur and nitrogen metabolism (73, 74), both of which are important for the coral holobiont and were proposed as BMC traits (41). In addition, three genes encoding proteins related to the nitrogen cycle (nitrite reductase, nitrate reductase, and urease protein) were found only in the genomes of Cobetia sp. BMC 6 (nitrite reductase) and Halomonas sp. BMC 7 (all three genes). BMCs associated with the biogeochemical cycling of nitrogen are potentially key members of tropical coral microbiomes, as coral reef systems are characterized by high primary productivity and low nutrient availability (75, 76). To thrive in oligotrophic environments, corals rely on the rapid assimilation and retention of nitrogen, which is the major limiting element of primary production in the ocean (77), whereas excessive amounts of nitrogen can also represent a threat to corals (78). Therefore, BMCs involved in nitrogen cycling may play an important role in alleviating nutrient limitation while also cycling excessive amounts of nitrogen compounds, helping to maintain host tolerance to nutrient-dense conditions such as those experienced during seasonal flood events (79) or helping to maintain a favorable nitrogen-phosphorus ratio (80).

A total of 710 genes with known functions were identified in the Halomonas sp. BMC 7 genome only (Fig. 6). Among those genes, 18 encode proteins were identified that may play a beneficial role for corals and *Symbiodiniaceae*, such as those related to nitrogen cycling and the siroheme synthetase protein mentioned above. Halomonas sp. BMC 7 harbors a gene encoding the protein flavohemoglobin, an oxidoreductase enzyme involved in the detoxification of nitric oxide (NO) in an aerobic process called the nitric oxide dioxide (NOD) reaction. This reaction uses O_2_ and NADPH to convert NO to nitrate and in this way protects cells from various harmful nitrogen compounds (81). It is worth highlighting that NO demonstrates a wide range of cellular toxicities that are known as “nitrosative stress,” an analogous term to oxidative stress. Although NO drives some of these toxicities directly, it is also increasingly clear that NO serves as a precursor molecule for a variety of reactive nitrogen species (RNS), such as dinitrogen trioxide (N_2_O_3_), nitrogen dioxide (NO_2•_), and peroxynitrite (ONOO_−_) (82, 83). These highly reactive RNS drive many biochemical reactions involving cellular proteins (e.g., nitration, nitrosylation, oxidation), DNA (e.g., deamination), and lipids (e.g., nitration and oxidation). The final product is usually loss of function for this affected biomolecule (84, 85). During heat stress, the amount of NO inside coral cells and *Symbiodiniaceae* increases, while excess NO reacts with O_2_ to form peroxynitrite, which in turn inhibits the transport of electrons in the mitochondrial electron transport chain (49, 86) and interrupts the host’s cellular respiration. Increased nitric oxide reductase and arginase-related genes (both involved in supporting cells in detoxifying nitric oxide from the host) have been previously correlated with diseased coral tissue (87). Therefore, any mechanism that reduces the excess of intracellular NO in the holobiont seems to be important for the health of corals.

Six genes encoding proteins related to the synthesis of B vitamins were found in Halomonas sp. BMC 7 only; these were *cobP*, *cobD*, and *cobQ* (related to cobalamin [B_12_] synthesis); the *thiL* gene related to thiamin (B_1_) synthesis; the *panC* gene related to pantothenate (B_5_) synthesis; and the *folC* gene related to folate (B_9_) synthesis. The production of B vitamins has been observed in other relationships between corals and bacteria and may be an important process for healthy coral functioning (88). The micronutrient cobalamin (vitamin B_12_) is involved in diverse metabolic pathways, including the generation of the antioxidants glutathione and DMSP (54). Thiamin (B_1_) plays a key role in intermediate carbon metabolism in algae and is a cofactor for several enzymes involved in the metabolism of primary carbohydrates and branched-chain amino acids (89). Folate (B_9_) is involved in the synthesis of *S*-adenosylmethionine, a methyl group donor in DNA methylation (90). Downregulation of genes involved in the pantothenate (B_5_) metabolic process has been observed in corals 10 h after heat stress, and this has been hypothesized to increase host susceptibility to pathogens due to downregulation of innate immune response (91). Together, these data reinforce that the production of B-complex vitamins is an important BMC characteristic. It is also possible that *Symbiodiniaceae* obtain B vitamins from the bacterial symbionts, as many algae do not have the capacity to produce these vitamins (89). Indeed, a recent study showed that the *Gammaproteobacteria* symbionts of healthy octocoral hold the genetic blueprint for the biosynthesis of all eight B vitamins (92).

Three genes encoding proteins related to siderophore biosynthesis were identified in Halomonas sp. BMC 7 only (*iucC*, *iucD*, and *iucA*). As previously mentioned, siderophores are organic compounds that capture iron from the environment; they bind and concentrate iron in bioavailable forms, allowing the absorption of this limiting micronutrient by many organisms. Another three genes related to protection against oxidative stress were also identified in the Halomonas sp. BMC 7 genome (*katG*, *oxyR*, and *ubiG*). An increase in temperature and irradiance results in increased production of ROS in corals and their endosymbiont photosynthetic algae symbionts, as discussed earlier, which overloads the defense mechanisms of both players and ultimately damages their cells so that it is one of the main drivers of coral bleaching (49–51).

A gene related to the DMSP degradation pathway was also identified in the Halomonas sp. BMC 7 genome only. The *dddP* gene encodes the protein dimethlysulfonioproprionate lyase, the enzyme that breaks down DMSP. The resulting decomposition products can act as antioxidants and protect algae from oxidative stress derived from photosynthesis (93, 94). Furthermore, Garren et al. (95) showed that the pathogen V. coralliilyticus displays DMSP chemotaxis to locate corals stressed by increasing temperature, wherefore the DMSP catabolism could be a mechanism that makes it difficult for the pathogen to localize the coral. In addition, a probiotic BMC consortium using a DMSP degrading strain has significantly reduced DMSP concentrations within the holobiont, which was also correlated with significant improvements on coral health and survivorship (10). Therefore, genes related to DMSP metabolism would fit in as an excellent target to search for BMC strains.

Many studies have highlighted the vital role of the microbiome and its metabolites in maintaining coral health and regulating ecosystem resilience (10, 28, 30). Microorganisms can mitigate anthropogenic impacts through their role in holobiont regulation (7, 20), as well as disrupting nutrient and energy flows in coral reefs (26, 96). It is therefore of great importance to understand the specific mechanisms of interactions between corals and their associated microbes. Although this study did not aim to show that BMCs have certain characteristics that that are not present in other members of the coral microbiome, our approach may facilitate the detection of microorganisms harboring these theoretically vital roles to the host health, which could therefore be tested as BMCs. The best way to validate the use of a BMC consortium as a means to mitigate damage caused by different stressors is to compare any health improvement with the use of a placebo treatment. Alternative microbial therapies, such as the use of heat-killed bacteria (also called postbiotics) can also help to understand the protective mechanisms provided by microbes (dead or alive). The use of microbes that were not selected by current screening methods can also generate data on unknown beneficial mechanisms.

In summary, our results indicate several possible theoretical ways in which BMCs could have acted to help corals during periods of stress (7). Although, as highlighted above, most of these characteristics are still theoretical, some of the putative beneficial genes have already been validated or shown to have differential expression during heat stress events in corals. For instance, metatranscriptome analyses have shown the differential expression of several genes in response to thermal stress in scleractinian corals, such as Mussismilia hispida, Siderastrea radians, Orbicella faveolata, and Pseudodiploria clivosa (10, 97). The results reveal that several genes previously suggested to be potentially beneficial to corals (and also detected in our genomes) were upregulated during stress, such as peroxidase genes related to protection against ROS (e.g., S. radians and O. faveolata), vitamin B biosynthesis (e.g., P. clivosa) (97), and host immune response elicited by shifts in the microbiome (10). Genes related to sulfur and nitrogen metabolism were also upregulated, with a higher expression in S. radians. This differential expression was mainly due to shifts in the microbiome, suggesting that the microbiome is primarily responsible for the upregulation of these genes in the studied corals (10, 97).

Considering the limitations and necessary improvements for the cultivation of coral-associated microbes (98), the use of *in silico* screenings can simultaneously accelerate the selection of cultured BMCs (since through genome analysis it is possible to quickly identify a greater variety of genes compared to PCR and biochemical/physiological assays) and improve our ability to culture alternative BMCs. This work provides an array of new gene targets to be incorporated into the BMC screening framework, while it also suggests potential alternative beneficial mechanisms involved in the microbial mitigation of coral bleaching.

## MATERIALS AND METHODS

### Genome sequencing of the BMC consortium.

Seven bacterial strains isolated from the coral P. damicornis by Rosado et al. (7) were originally selected, based on their putative beneficial traits (26), for a mesocosm experiment that demonstrated their beneficial effects on corals (7). These strains were previously identified by 16S rRNA gene sequencing as Pseudoalteromonas sp. (*n* = 5), Cobetia sp. (*n* = 1), and Halomonas sp. (*n* = 1).

For genome sequencing, the seven BMC strains were cultivated in Difco marine broth 2216 culture medium (Becton, Dickinson & Co. Sparks, USA) from their respective stocks kept in glycerol at −80°C. After 28 h under constant agitation at 26°C to 28°C, 1 mL of each strain was centrifuged at 13,000 × g for 2 min in 1.5-mL tubes; the supernatant was discarded, and the cell pellet was used for DNA extraction. For this purpose, a Wizard genomic DNA purification kit (Promega, Madison, WI, USA) was used following the manufacturer’s protocol. The genomic DNA was quantified using a Qubit 2.0 fluorometer double-stranded DNA (dsDNA) kit (Invitrogen, Carlsbad, CA, USA) and subjected to agarose gel electrophoresis (1%) to observe DNA quality. Genome sequencing of the seven strains was performed on an Illumina MiSeq platform with the NEBNext Ultra II FS DNA kit for library assembly and a MiSeq reagent kit v3 for flow cell, which generated between 1,405,296 and 2,187,986 paired end reads of 301 bp/genome for a total of 600 amplification cycles.

### Processing and assembly of the BMC genomes.

Raw sequence reads were quality-filtered using the Trimmomatic version 036 program (99) with a Phred score parameter value of 33 and the following arguments: sliding window size: 4; sliding window minimum quality: 20; head crop length: 5; leading minimum quality: 10; trailing minimum quality: 10; and minimum read length: 36. Quality of the filtered sequences was evaluated by the FastQC v. 0.11.5 program (http://www.bioinformatics.babraham.ac.uk/projects/fastqc/), followed by assembly of each genome using SPAdes v. 3.13.0 (100) with default parameters. Genome integrity and contamination values of the assembled genomes were calculated with the CheckM software version 1.0.18 (101), and sequences with a high contamination index were refined using the RefineM program version 0.0.23 (101, 102). Stretches with discrepant taxonomy sequences were removed using RefineM’s default settings, which allowed for coverage and contamination indices to be recalculated.

### Taxonomic attribution analysis.

Species-level classification of the seven genomes was performed using the FastANI tool (103) and the Genome-to-Genome Distance Calculator (GGDC). FastANI makes a pairwise comparison of complete genomes, but instead of aligning the entire sequence of the two genomes, it uses the Mashmap program (104). ANI values of >95% are widely used as a cutoff to determine the same-species status of genome sequences (103, 105, 106). The GGDC is a state-of-the-art *in silico* method for genome-to-genome comparison, reliably mimicking conventional DNA-DNA hybridization (DDH). A GGDC index with more than 70% similarity between two genome sequences indicates that both sequences belong to the same species. A DDH above 79% suggests that a pair of sequences belongs to the same subspecies (105, 107, 108). For the identification of BMCs 1, 2, 3, 4, and 5, previously identified by 16s rRNA gene analysis as Pseudoalteromonas sp. (7), the 17 genomes that formed a cluster with the BMCs (as assessed in a preliminary analysis in Fig. S1) were used as illustrated in the Pseudoalteromonas phylogenomic trees (Fig. 3; Fig. S1). For the identification of BMC 6, 10 genomes that formed a cluster with the BMC were used as illustrated in the Cobetia phylogenomic tree (Fig. 1). Finally, to identify BMC 7, three genomes that formed a cluster with the BMC were used as illustrated in the Halomonas phylogenomic tree (Fig. 2).

### Structural and functional annotation of genomes.

The seven genomes were annotated using Prokka (109), as well as the PATRIC Bacterial Bioinformatics Resource Center (110), which uses the RASTtk online server (111) with default parameters. The output files were visualized on PATRIC’s graphical user interface to build genomic maps of the studied bacteria and search for protein-coding genes indicating potentially beneficial characteristics for corals, such as genes related to nutrition and growth, mitigation of toxic compounds or stress, early coral development, and pathogen control (26, 41). The genes of interest were found by searching the subsystems generated by the RASTtk platform in PATRIC from the databases used in their default configurations.

### Assembly of phylogenomic trees.

To build phylogenomic trees, PATRIC’s codon tree method was used, which randomly selects up to 10 proteins to represent each genus-level family and combines them to form a single set of representatives in order to prevent cluster formation that is based upon the genus rather than protein similarity (112). Both protein (amino acid) and gene (nucleotide) sequences were used for each of the selected genes from the PATRIC global protein families (PGFams). Protein sequences were aligned using MUSCLE (113), and nucleotide-encoding gene sequences were aligned using the Codon_align function of BioPython (114). A file in phylip format was generated with the concatenated alignment of all proteins and nucleotides, and then a partitioned file for RAxML (115), describing the alignment in terms of proteins, was generated. Support values were generated using 100 rounds of the “Rapid” bootstrapping option (116) in RAxML. The phylogenomic tree was formed by concatenated open reading frames (ORFs) from 1,000 protein-coding genes (Fig. 1 to 3) and 100 protein-coding genes (Fig. S1) shared among all genomes present using the best protein model method found by RAxML (117). The result was generated in Newick format and visualized in iTOL version 6.5.3 (118), where the tree was assembled.

Three different trees were generated, one for each genus of BMC (Pseudoalteromonas, Cobetia, and Halomonas). The genomes of each genus that made up the trees were selected from the PATRIC database using different selection criteria, such as being reference genomes and/or complete genomes assembled in one contig of bacteria isolated from corals and/or aquatic environments. The host from which each strain was isolated was also added to the phylogenomic tree.

### Pangenomic analysis and gene categorization of BMC genomes.

The pangenome was generated separately for each genus of BMC (Pseudoalteromonas, Cobetia, and Halomonas) through the Roary program (119), using the default settings based on annotations made with Prokka. To generate each pangenome, sequences from the closest genomes of all BMCs based on taxonomic attribution results and phylogenetic trees were added to the analysis. More specifically, in the pangenome of the Pseudoalteromonas genus, 17 genomes of Pseudoalteromonas sp., which formed a clade with the BMC (Fig. S1), were added in addition to the 5 Pseudoalteromonas sp. BMCs (Fig. 3), totaling 22 genomes used for this pangenome analysis. For the Cobetia sp. pangenome, 10 genomes were used in addition to the Cobetia sp. BMC 6, totaling 11 genomes. The selection of these 10 genomes was based on the results of the taxonomic analysis of the ANI (Table S2) and on the clade formed with BMC 6 in the phylogenomic tree (Fig. 1). Finally, for the Halomonas sp. pangenome, 3 genomes were used (H. taeanensis BH539, H. taeanensis USBA-857, and Halomonas sp. YLGW01) in addition to the Halomonas sp. BMC 7, totaling 4 genomes. The selection of these 3 genomes was also based on the results provided by the taxonomic analysis of the ANI (Table S2) and on the clade formed with BMC 7 in the phylogenomic tree (Fig. 2). All genomes used for comparison with the BMC genomes were obtained from the PATRIC database (112). The pangenome figures were created using the Anvi’o visualization platform (120).

The categorization of genes used the results provided by the pangenomic analysis. Singleton genes from each of the BMCs were used for the purpose of analyzing which types of genes were unique to each BMC and to verify their potential to promote any benefit to the host. We define singleton genes as genes that are present in only one strain of the BMC or genes that were shared only among the BMCs but that do not appear in the genomes of other members of the genus included in the analyses.

### Data availability.

The complete genome sequencing data, including raw sequence reads, genome assemblies, and annotations of the BMC strains (BMC 1, BMC 2, BMC 3, BMC 4, BMC 5, BMC 6, and BMC 7) used in this study were submitted to NCBI, GenBank under the BioProject accession PRJNA638634 and BioSample accession numbers SAMN15198640 to SAMN15198646. The genomes used for comparison are available in Table S3.

10.1128/msystems.00921-22.4 TABLE S3 Genomes used in the genomic and pangenome comparisons performed in this study. Download Table S3, DOCX file, 0.01 MB. Copyright © 2023 Rosado et al. 2023 Rosado et al. https://creativecommons.org/licenses/by/4.0/ This content is distributed under the terms of the Creative Commons Attribution 4.0 International license.

## Supplementary Material

Reviewer comments

## References

[1] Maloy S, Moran MA, Mulholland MR, Sosik HM, Spear JR. 2017. Microbes and climate change: report on an American Academy of Microbiology and American Geophysical Union Colloquium held in Washington, DC, in March 2016, American Society for Microbiology, Washington, DC. 30063309

[2] Sharp K, Ritchie KB. 2012. Multi-partner interactions in corals in the face of climate change. Biol Bull 223:66–77. doi:10.1086/BBLv223n1p66. 22983033

[3] Zhang Y, Yang Q, Ling J, Long L, Huang H, Yin J, Wu M, Tang X, Lin X, Zhang Y, Dong J. 2021. Shifting the microbiome of a coral holobiont and improving host physiology by inoculation with a potentially beneficial bacterial consortium. BMC Microbiol 21:1–14. doi:10.1186/s12866-021-02167-5. 33910503 PMC8082877

[4] Alagely A, Krediet CJ, Ritchie KB, Teplitski M. 2011. Signaling-mediated cross-talk modulates swarming and biofilm formation in a coral pathogen *Serratia marcescens*. ISME J 5:1609–1620. doi:10.1038/ismej.2011.45. 21509042 PMC3176518

[5] Krediet CJ, Ritchie KB, Paul VJ, Teplitski M. 2013. Coral-associated micro-organisms and their roles in promoting coral health and thwarting diseases. Proc Biol Sci 280:20122328. doi:10.1098/rspb.2012.2328. 23363627 PMC3574386

[6] Welsh RM, Rosales SM, Zaneveld JR, Payet JP, McMinds R, Hubbs SL, Thurber RLV. 2017. Alien vs. predator: bacterial challenge alters coral microbiomes unless controlled by Halobacteriovorax predators. PeerJ 5:e3315. doi:10.7717/peerj.3315. 28584701 PMC5455293

[7] Rosado PM, Leite DCA, Duarte GAS, Chaloub RM, Jospin G, Nunes da Rocha UP, Saraiva JP, Dini-Andreote F, Eisen JA, Bourne DG, Peixoto RS. 2019. Marine probiotics: increasing coral resistance to bleaching through microbiome manipulation. ISME J 13:921–936. doi:10.1038/s41396-018-0323-6. 30518818 PMC6461899

[8] Williams AD, Brown BE, Putchim L, Sweet MJ. 2015. Age-related shifts in bacterial diversity in a reef coral. PLoS One 10:e0144902. doi:10.1371/journal.pone.0144902. 26700869 PMC4689413

[9] Nair S, Abraham J. 2019. Coral reef microbiota and its role in marine ecosystem sustainability, p 453–478. *In* Microbial Interventions in Agriculture and Environment. Springer International Publishing, Cham, Switzerland.

[10] Santoro EP, Borges RM, Espinoza JL, Freire M, Messias CS, Villela HDM, Pereira ML, Vilela CLS, Rosado JG, Cardoso PM, Rosado PM, Assis JM, Duarte GAS, Perna G, Rosado AS, Macrae A, Dupont CL, Nelson KE, Sweet MJ, Voolstra CR, Peixoto RS. 2021. Coral microbiome manipulation elicits metabolic and genetic restructuring to mitigate heat stress and evade mortality. Sci Adv 7:eabg3088. doi:10.1126/sciadv.abg3088. 34389536 PMC8363143

[11] Hernandez-Agreda A, Leggat W, Bongaerts P, Ainsworth TD. 2016. The microbial signature provides insight into the mechanistic basis of coral success across reef habitats. mBio 7:e00560-16. doi:10.1128/mBio.00560-16. 27460792 PMC4981706

[12] Petersen C, Round JL. 2014. Defining dysbiosis and its influence on host immunity and disease. Cell Microbiol 16:1024–1033. doi:10.1111/cmi.12308. 24798552 PMC4143175

[13] Egan S, Gardiner M. 2016. Microbial dysbiosis: rethinking disease in marine ecosystems. Front Microbiol 7:991. doi:10.3389/fmicb.2016.00991. 27446031 PMC4914501

[14] Meron D, Rodolfo-Metalpa R, Cunning R, Baker AC, Fine M, Banin E. 2012. Changes in coral microbial communities in response to a natural pH gradient. ISME J 6:1775–1785. doi:10.1038/ismej.2012.19. 22437157 PMC3498918

[15] Grottoli AG, Dalcin Martins P, Wilkins MJ, Johnston MD, Warner ME, Cai WJ, Melman TF, Hoadley KD, Pettay DT, Levas S, Schoepf V. 2018. Coral physiology and microbiome dynamics under combined warming and ocean acidification. PLoS One 13:e0191156. doi:10.1371/journal.pone.0191156. 29338021 PMC5770069

[16] Epstein HE, Torda G, van Oppen MJH. 2019. Relative stability of the *Pocillopora acuta* microbiome throughout a thermal stress event. Coral Reefs 38:373–386. doi:10.1007/s00338-019-01783-y.

[17] Salerno JL, Reineman DR, Gates RD, Rappé MS. 2011. The effect of a sublethal temperature elevation on the structure of bacterial communities associated with the coral *porites compressa*. J Mar Biol 2011:969173. doi:10.1155/2011/969173.

[18] Santos HF, Carmo FL, Duarte GAS, Dini-Andreote F, Castro CB, Rosado AS, van Elsas JD, Peixoto RS. 2014. Climate change affects key nitrogen-fixing bacterial populations on coral reefs. ISME J 8:2272–2279. doi:10.1038/ismej.2014.70. 24830827 PMC4992079

[19] Leite DCA, Salles JF, Calderon EN, Castro CB, Bianchini A, Marques JA, van Elsas JD, Peixoto RS. 2018. Coral bacterial-core abundance and network complexity as proxies for anthropogenic pollution. Front Microbiol 9:833. doi:10.3389/fmicb.2018.00833. 29755445 PMC5934943

[20] Santos HF, Duarte GAS, Rachid CT, Chaloub RM, Calderon EN, Marangoni LF, Bianchini A, Nudi AH, Carmo FL, van Elsas JD, Rosado AS, Castro CB, Peixoto RS. 2015. Impact of oil spills on coral reefs can be reduced by bioremediation using probiotic microbiota. Sci Rep 5:18268. doi:10.1038/srep18268. 26658023 PMC4677405

[21] Silva DP, Villela HDM, Santos HF, Duarte GAS, Ribeiro RJ, Ghizelini AM, Vilela CLS, Rosado PM, Fazolato CS, Santoro EP, Carmo FL, Ximenes DS, Soriano AU, Rachid CTCC, Thurber RLV, Peixoto RS. 2021. Multi-domain probiotic consortium as an alternative to chemical remediation of oil spills at coral reefs and adjacent sites. Microbiome 9:118. doi:10.1186/s40168-021-01041-w. 34020712 PMC8138999

[22] Bourne DG, Iida Y, Uthicke S, Smith- Keune C. 2008. Changes in coral-associated microbial communities during a bleaching event. ISME J 2:350–363. doi:10.1038/ismej.2007.112. 18059490

[23] Ziegler M, Grupstra CGB, Barreto MM, Eaton M, BaOmar J, Zubier K, Al-Sofyani A, Turki AJ, Ormond R, Voolstra CR. 2019. Coral bacterial community structure responds to environmental change in a host-specific manner. Nat Commun 10:3092. doi:10.1038/s41467-019-10969-5. 31300639 PMC6626051

[24] Tracy AM, Koren O, Douglas N, Weil E, Harvell CD. 2015. Persistent shifts in Caribbean coral microbiota are linked to the 2010 warm thermal anomaly. Environ Microbiol Rep 7:471–479. doi:10.1111/1758-2229.12274. 25683053

[25] Torda G, Donelson JM, Aranda M, Barshis DJ, Bay L, Berumen ML, Bourne DG, Cantin N, Foret S, Matz M, Miller DJ, Moya A, Putnam HM, Ravasi T, van Oppen MJH, Thurber RV, Vidal-Dupiol J, Voolstra CR, Watson SA, Whitelaw E, Willis BL, Munday PL. 2017. Rapid adaptive responses to climate change in corals. Nat Clim Chang 7:627–636. doi:10.1038/nclimate3374.

[26] Peixoto RS, Rosado PM, Leite DCA, Rosado AS, Bourne DG. 2017. Beneficial microorganisms for corals (BMC): proposed mechanisms for coral health and resilience. Front Microbiol 8:341. 28326066 10.3389/fmicb.2017.00341 PMC5339234

[27] Peixoto RS, Harkins DM, Nelson KE. 2021. Advances in microbiome research for animal health. Annu Rev Anim Biosci 9:289–311. doi:10.1146/annurev-animal-091020-075907. 33317323

[28] Peixoto RS, Voolstra CR, Sweet M, Duarte CM, Carvalho S, Villela HDM, Lunshof JE, Gram L, Woodhams DC, Walter J, Roik A, Hentschel U, Thurber RV, Daisley B, Ushijima B, Daffonchio D, Costa R, Keller-Costa T, Bowman JS, Rosado AS, Reid G, Mason CE, Walke JB, Thomas T, Berg G. 2022. Harnessing the microbiome to prevent global biodiversity loss. Nat Microbiol 7:1726–1735. doi:10.1038/s41564-022-01173-1. 35864220

[29] Boilard A, Dube CE, Gruet C, Merciere A, Hernandez-Agreda A, Derome N. 2020. Defining coral bleaching as a microbial dysbiosis within the coral holobiont. Microorganisms 8:1682. doi:10.3390/microorganisms8111682. 33138319 PMC7692791

[30] Voolstra CR, Suggett DJ, Peixoto RS, Parkinson JE, Quigley KM, Silveira CB, Sweet M, Muller EM, Barshis DJ, Bourne DG, Aranda M. 2021. Extending the natural adaptive capacity of coral holobionts. Nat Rev Earth Environ 2:747–762. doi:10.1038/s43017-021-00214-3.

[31] Webster NS, Reusch TBH. 2017. Microbial contributions to the persistence of coral reefs. ISME J 11:2167–2174. doi:10.1038/ismej.2017.66. 28509908 PMC5607359

[32] Leite DCA, Leão P, Garrido AG, Lins U, Santos HF, Pires DO, Castro CB, van Elsas JD, Zilberberg C, Rosado AS, Peixoto RS. 2017. Broadcast spawning coral *Mussismilia hispida* can vertically transfer its associated bacterial core. Front Microbiol 8:176. doi:10.3389/fmicb.2017.00176. 28223979 PMC5293827

[33] Quigley KM, Willis BL, Kenkel CD. 2019. Transgenerational inheritance of shuffled symbiont communities in the coral *Montipora digitata*. Sci Rep 9:13328. doi:10.1038/s41598-019-50045-y. 31527788 PMC6746730

[34] Barno AR, Villela HDM, Aranda M, Thomas T, Peixoto RS. 2021. Host under epigenetic control: a novel perspective on the interaction between microorganisms and corals. Bioessays 43:2100068. doi:10.1002/bies.202100068. 34463364

[35] West AG, Waite DW, Deines P, Bourne DG, Digby A, McKenzie VJ, Taylor MW. 2019. The microbiome in threatened species conservation. Biol Conserv 229:85–98. doi:10.1016/j.biocon.2018.11.016.

[36] Peixoto RS, Sweet M, Bourne DG. 2019. Customized medicine for corals. Front Mar Sci 6:686. doi:10.3389/fmars.2019.00686.

[37] Damjanovic K, van Oppen MJH, Menéndez P, Blackall LL. 2019. Experimental inoculation of coral recruits with marine bacteria indicates scope for microbiome manipulation in *Acropora tenuis* and *Platygyra daedalea*. Front Microbiol 10:1702. doi:10.3389/fmicb.2019.01702. 31396197 PMC6668565

[38] Morgans CA, Hung JY, Bourne DG, Quigley KM. 2020. *Symbiodiniaceae* probiotics for use in bleaching recovery. Restor Ecol 28:282–288. doi:10.1111/rec.13069.

[39] Doering T, Wall M, Putchim L, Rattanawongwan T, Schroeder R, Hentschel U, Roik A. 2021. Towards enhancing coral heat tolerance: a “microbiome transplantation” treatment using inoculations of homogenized coral tissues. Microbiome 9:102. doi:10.1186/s40168-021-01053-6. 33957989 PMC8103578

[40] Reshef L, Koren O, Loya Y, Zilber-Rosenberg I, Rosenberg E. 2006. The coral probiotic hypothesis. Environ Microbiol 8:2068–2073. doi:10.1111/j.1462-2920.2006.01148.x. 17107548

[41] Peixoto RS, Sweet M, Villela HDM, Cardoso P, Thomas T, Voolstra CR, Hoj L, Bourne DG. 2021. Coral probiotics: premise, promise, prospects. Annu Rev Anim Biosci 9:265–288. 33321044 10.1146/annurev-animal-090120-115444

[42] Matthews JL, Raina JB, Kahlke T, Seymour JR, van Oppen MJH, Suggett DJ. 2020. *Symbiodiniaceae*-bacteria interactions: rethinking metabolite exchange in reef-building corals as multi-partner metabolic networks. Environ Microbiol 22:1675–1687. doi:10.1111/1462-2920.14918. 31943674

[43] Matsuyama H, Sawazaki K, Minami H, Kasahara H, Horikawa K, Yumoto I. 2014. *Pseudoalteromonas shioyasakiensis* sp. nov., a marine polysaccharide-producing bacterium. Int J Syst Evol Microbiol 64:101–106. doi:10.1099/ijs.0.055558-0. 24021728

[44] Arahal DR, Castillo AM, Ludwig W, Schleifer KH, Ventosa A. 2002. Proposal of *Cobetia marina* gen. nov., comb. nov., within the family *Halomonadaceae*, to include the species *Halomonas marina*. System Appl Microbiol 25:207–211. doi:10.1078/0723-2020-00113. 12353874

[45] Maric L, Cleenwerck I, Accetto T, Vandamme P, Trcek J. 2020. Description of *Komagataeibacter melaceti* sp. nov. and *Komagataeibacter melomenusus* sp. nov. isolated from apple cider vinegar. Microorganisms 8:1178. doi:10.3390/microorganisms8081178. 32756518 PMC7465234

[46] Hayyan M, Hashim MA, Al Nashef IM. 2016. Superoxide ion: generation and chemical implications. Chem Rev 116:3029–3085. doi:10.1021/acs.chemrev.5b00407. 26875845

[47] Njålsson R, Norgren S. 2005. Physiological and pathological aspects of GSH metabolism. Acta Paediatr 94:132–137. doi:10.1111/j.1651-2227.2005.tb01878.x. 15981742

[48] Barycki JJ, Asard H, Stone JM, Wilson MA, Banerjee R, Becker DF. 2007. Antioxidant molecules and redox cofactors, p 11–47. *In* Redox biochemistry. John Wiley & Sons, Inc., Hoboken, NJ.

[49] Weis VM, Davy SK, Hoegh-Guldberg O, Rodriguez-Lanetty M, Pringle JR. 2008. Cell biology in model systems as the key to understanding corals. Trends Ecol Evol 23:369–376. doi:10.1016/j.tree.2008.03.004. 18501991

[50] Roberty S, Furla P, Plumier JC. 2016. Differential antioxidant response between two *Symbiodinium* species from contrasting environments. Plant Cell Environ 39:2713–2724. doi:10.1111/pce.12825. 27577027

[51] Szabó M, Larkum AWD, Vass I. 2020. A review: the role of reactive oxygen species in mass coral bleaching, p 459–488. *In* Larkum AWD, Grossmann AR, Raven JA (ed), Photosynthesis in algae: biochemical and physiological mechanisms. Springer International Publishing, Cham, Switzerland.

[52] Duarte GAS, Villela HDM, Deocleciano M, Silva D, Barno A, Cardoso PM, Vilela CLS, Rosado P, Messias CSMA, Chacon MA, Santoro EP, Olmedo DB, Szpilman M, Rocha LA, Sweet M, Peixoto RS. 2020. Heat waves are a major threat to turbid coral reefs in Brazil. Front Mar Sci 7:179. doi:10.3389/fmars.2020.00179.

[53] Hughes TP, Kerry JT, Alvarez-Noriega M, Alvarez-Romero JG, Anderson KD, Baird AH, Babcock RC, Beger M, Bellwood DR, Berkelmans R, Bridge TC, Butler IR, Byrne M, Cantin NE, Comeau S, Connolly SR, Cumming GS, Dalton SJ, Diaz-Pulido G, Eakin CM, Figueira WF, Gilmour JP, Harrison HB, Heron SF, Hoey AS, Hobbs JA, Hoogenboom MO, Kennedy EV, Kuo C, Lough JM, Lowe RJ, Liu G, McCulloch MT, Malcolm HA, McWilliam MJ, Pandolfi JM, Pears RJ, Pratchett MS, Schoepf V, Simpson T, Skirving WJ, Sommer B, Torda G, Wachenfeld DR, Willis BL, Wilson KS. 2017. Global warming and recurrent mass bleaching of corals. Nature 543:373–377. doi:10.1038/nature21707. 28300113

[54] Croft MT, Lawrence AD, Raux-Deery E, Warren MJ, Smith AG. 2005. Algae acquire vitamin B_12_ through a symbiotic relationship with bacteria. Nature 438:90–93. doi:10.1038/nature04056. 16267554

[55] Sweet M, Villela H, Keller-Costa T, Costa R, Romano S, Bourne DG, Cardenas A, Huggett MJ, Kerwin AH, Kuek F, Medina M, Meyer JL, Muller M, Pollock FJ, Rappe MS, Sere M, Sharp KH, Voolstra CR, Zaccardi N, Ziegler M, Peixoto RS. 2021. Insights into the cultured bacterial fraction of corals. mSystems 6:e01249-20. doi:10.1128/mSystems.01249-20. 34156291 PMC8269258

[56] Behrenfeld MJ, Westberry TK, Boss ES, O’Malley RT, Siegel DA, Wiggert JD, Franz BA, McClain CR, Feldman GC, Doney SC, Moore JK, Dall’Olmo G, Milligan AJ, Lima I, Mahowald N. 2008. Satellite-detected fluorescence reveals global physiology of ocean phytoplankton. Biogeosci Discuss 5:4235–4270.

[57] Hopkinson BM, Morel FM. 2009. The role of siderophores in iron acquisition by photosynthetic marine microorganisms. Biometals 22:659–669. doi:10.1007/s10534-009-9235-2. 19343508

[58] Amin SA, Green DH, Hart MC, Küpper FC, Sunda WG, Carrano CJ. 2009. Photolysis of iron–siderophore chelates promotes bacterial–algal mutualism. Proc Natl Acad Sci USA 106:17071–17076. doi:10.1073/pnas.0905512106. 19805106 PMC2761308

[59] Lawson CA, Raina J, Kahlke T, Seymour JR, Suggett DJ. 2018. Defining the core microbiome of the symbiotic dinoflagellate, *Symbiodinium*. Environ Microbiol Rep 10:7–11. doi:10.1111/1758-2229.12599. 29124895

[60] Bownik A, Stępniewska Z, Skowroński T. 2014. Protective effects of ectoine on heat stressed *Daphnia magna*. J Comp Physiol B 184:961–976. doi:10.1007/s00360-014-0860-x. 25223383 PMC4234998

[61] Chen THH, Murata N. 2002. Enhancement of tolerance of abiotic stress by metabolic engineering of betaines and other compatible solutes. Curr Opin Plant Biol 5:250–257. doi:10.1016/s1369-5266(02)00255-8. 11960744

[62] Burg MB, Ferraris JD. 2008. Intracellular organic osmolytes: function and regulation. J Biol Chem 283:7309–7313. doi:10.1074/jbc.R700042200. 18256030 PMC2276334

[63] Ngugi DK, Ziegler M, Duarte CM, Voolstra CR. 2020. Genomic blueprint of glycine betaine metabolism in coral metaorganisms and their contribution to reef nitrogen budgets. Iscience 23:101120–101121. doi:10.1016/j.isci.2020.101120. 32438323 PMC7240134

[64] Hill RW, Li C, Jones AD, Gunn JP, Frade PR. 2010. Abundant betaines in reef-building corals and ecological indicators of a photoprotective role. Coral Reefs 29:869–880. doi:10.1007/s00338-010-0662-x.

[65] Miethke M, Klotz O, Linne U, May JJ, Beckering CL, Marahiel MA. 2006. Ferri-bacillibactin uptake and hydrolysis in *Bacillus subtilis*. Mol Microbiol 61:1413–1427. doi:10.1111/j.1365-2958.2006.05321.x. 16889643

[66] Wang Q, Liu Q, Ma Y, Rui H, Zhang Y. 2007. LuxO controls extracellular protease, haemolytic activities and siderophore production in fish pathogen *Vibrio alginolyticus*. J Appl Microbiol 103:1525–1534. doi:10.1111/j.1365-2672.2007.03380.x. 17953563

[67] Cawoy H, Debois D, Franzil L, de Pauw E, Thonart P, Ongena M. 2015. Lipopeptides as main ingredients for inhibition of fungal phytopathogens by *Bacillus subtilis/amyloliquefaciens*. Microb Biotechnol 8:281–295. doi:10.1111/1751-7915.12238. 25529983 PMC4353342

[68] Huang X, Lu Z, Zhao H, Bie X, Lü F, Yang S. 2006. Antiviral activity of antimicrobial lipopeptide from *Bacillus subtilis* fmbj against pseudorabies virus, porcine parvovirus, newcastle disease virus and infectious bursal disease virus *in vitro*. Int J Pept Res Ther 12:373–377. doi:10.1007/s10989-006-9041-4.

[69] Raaijmakers JM, de Bruijn I, Nybroe O, Ongena M. 2010. Natural functions of lipopeptides from *Bacillus* and *Pseudomonas*: more than surfactants and antibiotics. FEMS Microbiol Rev 34:1037–1062. doi:10.1111/j.1574-6976.2010.00221.x. 20412310

[70] Vahidinasab M, Lilge L, Reinfurt A, Pfannstiel J, Henkel M, Heravi KM, Hausmann R. 2020. Construction and description of a constitutive plipastatin mono-producing *Bacillus subtilis*. Microb Cell Fact 19:205. doi:10.1186/s12934-020-01468-0. 33167976 PMC7654001

[71] Schwarz G, Mendel RR. 2006. Molybdenum cofactor biosynthesis and molybdenum enzymes. Annu Rev Plant Biol 57:623–647. doi:10.1146/annurev.arplant.57.032905.105437. 16669776

[72] Paoli M, Marles-Wright J, Smith A. 2002. Structure–function relationships in heme-proteins. DNA Cell Biol 21:271–280. doi:10.1089/104454902753759690. 12042067

[73] Stroupe ME, Leech HK, Daniels DS, Warren M, Getzoff ED. 2003. CysG structure reveals tetrapyrrole-binding features and novel regulation of siroheme biosynthesis. Nat Struct Biol 10:1064–1073. doi:10.1038/nsb1007. 14595395

[74] Dailey HA, Dailey TA, Gerdes S, Jahm D, Jahn M, O’Brian MR, Warren MJ. 2017. Prokaryotic heme biosynthesis: multiple pathways to a common essential product. Microb Mol Biol Rev 81:e00048-16. doi:10.1128/MMBR.00048-16. PMC5312243 28123057

[75] Falkowski PG, Dubinsky Z, Muscatine L, McCloskey L. 1993. Population control in symbiotic corals. Bioscience 43:606–611. doi:10.2307/1312147.

[76] Glaze TD, Erler DV, Siljanen HMP. 2022. Microbially facilitated nitrogen cycling in tropical corals. ISME J 16:68–77. doi:10.1038/s41396-021-01038-1. 34226659 PMC8692614

[77] Tyrrell T. 1999. The relative influences of nitrogen and phosphorus on oceanic primary production. Nature 400:525–531. doi:10.1038/22941.

[78] Morris LA, Voolstra CR, Quigley KM, Bourne DG, Bay LK. 2019. Nutrient availability and metabolism affect the stability of coral–*Symbiodiniaceae* symbioses. Trends Microbiol 27:678–689. doi:10.1016/j.tim.2019.03.004. 30987816

[79] Rädecker N, Pogoreutz C, Voolstra CR, Wiedenmann J, Wild C. 2015. Nitrogen cycling in corals: the key to understanding holobiont functioning? Trends Microbiol 23:490–497. doi:10.1016/j.tim.2015.03.008. 25868684

[80] Tilstra A, El-Khaled YC, Roth F, Rädecker N, Pogoreutz C, Voolstra CR, Wild C. 2019. Denitrification aligns with N2 fixation in Red Sea corals. Sci Rep 9:19460. doi:10.1038/s41598-019-55408-z. 31857601 PMC6923481

[81] Gardner AM, Gardner PR. 2002. Flavohemoglobin detoxifies nitric oxide in aerobic, but not anaerobic. J Biol Chem 277:8166–8171. doi:10.1074/jbc.M110470200. 11751864

[82] Lancaster JR, Jr. 2006. Nitroxidative, nitrosative, and nitrative stress: kinetic predictions of reactive nitrogen species chemistry under biological conditions. Chem Res Toxicol 19:1160–1174. doi:10.1021/tx060061w. 16978020

[83] Hill BG, Dranka BP, Bailey SM, Lancaster JR, Jr, Darley-Usmar VM. 2010. What part of NO don’t you understand? Some answers to the cardinal questions in nitric oxide biology. J Biol Chem 285:19699–19704. doi:10.1074/jbc.R110.101618. 20410298 PMC2888379

[84] Forrester MT, Stamler JS. 2007. A classification scheme for redox-based modifications of proteins. Am J Respir Cell Mol Biol 36:135–137. doi:10.1165/rcmb.2006-001ED. 17227880

[85] Pacher P, Beckman JS, Liaudet L. 2007. Nitric oxide and peroxynitrite in health and disease. Physiol Rev 87:315–424. doi:10.1152/physrev.00029.2006. 17237348 PMC2248324

[86] Oakley CA, Durand E, Wilkinson SP, Peng L, Weis VM, Grossman AR, Davy SK. 2017. Thermal shock induces host proteostasis disruption and endoplasmic reticulum stress in the model symbiotic cnidarian Aiptasia. J Proteome Res 16:2121–2134. doi:10.1021/acs.jproteome.6b00797. 28474894

[87] Keller-Costa T, Lago-Leston A, Saraiva JP, Toscan R, Silva SG, Goncalves J, Cox CJ, Kyrpides N, Nunes da Rocha U, Costa R. 2021. Metagenomic insights into the taxonomy, function, and dysbiosis of prokaryotic communities in octocorals. Microbiome 9:72. doi:10.1186/s40168-021-01031-y. 33766108 PMC7993494

[88] Agostini S, Suzuki Y, Higuchi T, Casareto BE, Yoshinaga K, Nakano Y, Fujimura H. 2012. Biological and chemical characteristics of the coral gastric cavity. Coral Reefs 31:147–156. doi:10.1007/s00338-011-0831-6.

[89] Tang YZ, Koch F, Gobler CJ. 2010. Most harmful algal bloom species are vitamin B_1_ and B_12_ auxotrophs. Proc Natl Acad Sci USA 107:20756–20761. doi:10.1073/pnas.1009566107. 21068377 PMC2996436

[90] Crider KS, Yang TP, Berry RJ, Bailey LB. 2012. Folate and DNA methylation: a review of molecular mechanisms and the evidence forfolate’s role. Adv Nutr 3:21–38. doi:10.3945/an.111.000992. 22332098 PMC3262611

[91] Hou J, Xu T, Su D, Wu Y, Cheng L, Wang J, Zhou Z, Wang Y. 2018. RNA-Seq reveals extensive transcriptional response to heat stress in the stony coral *Galaxea fascicularis*. Front Genet 9:37. doi:10.3389/fgene.2018.00037. 29487614 PMC5816741

[92] Keller-Costa T, Kozma L, Silva SG, Toscan R, Gonçalves J, Lago-Lestón A, Kyrpides NC, Nunes da Rocha U, Costa R. 2022. Metagenomics-resolved genomics provides novel insights into chitin turnover, metabolic specialization, and niche partitioning in the octocoral microbiome. Microbiome 10:151. doi:10.1186/s40168-022-01343-7. 36138466 PMC9502895

[93] Sunda W, Kieber DJ, Kiene RP, Huntsman S. 2002. An antioxidant function for DMSP and DMS in marine algae. Nature 418:317–320. doi:10.1038/nature00851. 12124622

[94] Deschaseaux ESM, Jones GB, Deseo MA, Shepherd KM, Kiene RP, Swan HB, Harrison PL, Eyre BD. 2014. Effects of environmental factors on dimethylated sulfur compounds and their potential role in the antioxidant system of the coral holobiont. Limnol Oceanogr 59:758–768. doi:10.4319/lo.2014.59.3.0758.

[95] Garren M, Son K, Raina J-B, Rusconi R, Menolascina F, Shapiro OH, Tout J, Bourne DG, Seymour JR, Stocker R. 2014. A bacterial pathogen uses dimethylsulfoniopropionate as a cue to target heat-stressed corals. ISME J 8:999–1007. doi:10.1038/ismej.2013.210. 24335830 PMC3996689

[96] Bourne DG, Morrow KM, Webster NS. 2016. Insights into the coral microbiome: underpinning the health and resilience of reef ecosystems. Annu Rev Microbiol 70:317–340. doi:10.1146/annurev-micro-102215-095440. 27482741

[97] Avila-Magana V, Kamel B, DeSalvo M, Gomez-Campo K, Enriquez S, Kitano H, Rohlfs RV, Iglesias-Prieto R, Medina M. 2021. Elucidating gene expression adaptation of phylogenetically divergent coral holobionts under heat stress. Nat Commun 12:5731. doi:10.1038/s41467-021-25950-4. 34593802 PMC8484447

[98] Schultz J, Modolon F, Rosado AS, Voolstra CR, Sweet M, Peixoto RS. 2022. Methods and strategies to uncover coral-associated microbial dark matter. mSystems 7:e00367-22. doi:10.1128/msystems.00367-22. 35862824 PMC9426423

[99] Bolger AM, Lohse M, Usadel B. 2014. Trimmomatic: a flexible trimmer for Illumina sequence data. Bioinformatics 30:2114–2120. doi:10.1093/bioinformatics/btu170. 24695404 PMC4103590

[100] Bankevich A, Nurk S, Antipov D, Gurevich AA, Dvorkin M, Kulikov AS, Lesin VM, Nikolenko SI, Pham S, Prjibelski AD, Pyshkin AV, Sirotkin AV, Vyahhi N, Tesler G, Alekseyev MA, Pevzner PA. 2012. SPAdes: a new genome assembly algorithm and its applications to single-cell sequencing. J Comput Biol 19:455–477. doi:10.1089/cmb.2012.0021. 22506599 PMC3342519

[101] Parks DH, Imelfort M, Skennerton CT, Hugenholtz P, Tyson GW. 2015. CheckM: assessing the quality of microbial genomes recovered from isolates, single cells, and metagenomes. Genome Res 25:1043–1055. doi:10.1101/gr.186072.114. 25977477 PMC4484387

[102] Parks DH, Rinke C, Chuvochina M, Chaumeil PA, Woodcroft BJ, Evans PN, Hugenholtz P, Tyson GW. 2017. Recovery of nearly 8,000 metagenome-assembled genomes substantially expands the tree of life. Nat Microbiol 2:1533–1542. doi:10.1038/s41564-017-0012-7. 28894102

[103] Jain C, Rodriguez-R LM, Phillippy AM, Konstantinidis KT, Aluru S. 2018. High-throughput ANI analysis of 90K prokaryotic genomes reveals clear species boundaries. Nat Commun 9:5114. doi:10.1038/s41467-018-07641-9. 30504855 PMC6269478

[104] Jain C, Koren S, Dilthey A, Phillippy AM, Aluru S. 2018. A fast adaptive algorithm for computing whole-genome homology maps. Bioinformatics 34:i748–i756. doi:10.1093/bioinformatics/bty597. 30423094 PMC6129286

[105] Goris J, Konstantinidis KT, Klappenbach JA, Coenye T, Vandamme P, Tiedje JM. 2007. DNA-DNA hybridization values and their relationship to whole genome sequence similarities. Int J Syst Evol Microbiol 57:81–91. doi:10.1099/ijs.0.64483-0. 17220447

[106] Konstantinidis KT, Tiedje JM. 2005. Genomic insights that advance the species definition for prokaryotes. Proc Natl Acad Sci USA 102:2567–2572. doi:10.1073/pnas.0409727102. 15701695 PMC549018

[107] Richter M, Rossello-Mora R. 2009. Shifting the genomic gold standard for the prokaryotic species definition. Proc Natl Acad Sci USA 106:19126–19131. doi:10.1073/pnas.0906412106. 19855009 PMC2776425

[108] Meier-Kolthoff JP, Auch AF, Klenk HP, Göker M. 2013. Genome sequence-based species delimitation with confidence intervals and improved distance functions. BMC Bioinformatics 14:60–14. doi:10.1186/1471-2105-14-60. 23432962 PMC3665452

[109] Seemann T. 2014. Prokka: rapid prokaryotic genome annotation. Bioinformatics 30:2068–2069. doi:10.1093/bioinformatics/btu153. 24642063

[110] Wattam AR, Abraham D, Dalay O, Disz TL, Driscoll T, Gabbard JL. 2014. PATRIC, the bacterial bioinformatics database and analysis resource. Nucleic Acids Res 42:581–591. 10.1093/nar/gkt1099 PMC3965095 24225323

[111] Brettin T, Davis JJ, Disz T, Edwards RA, Gerdes S, Olsen GJ, Olson R, Overbeek R, Parrello B, Pusch GD, Shukla M, Thomason JA, III, Stevens R, Vonstein V, Wattam AR, Xia F. 2015. RASTtk: a modular and extensible implementation of the RAST algorithm for building custom annotation pipelines and annotating batches of genomes. Sci Rep 5:8365. doi:10.1038/srep08365. 25666585 PMC4322359

[112] Davis JJ, Wattam AR, Aziz RK, Brettin T, Butler R, Butler RM, Chlenski P, Conrad N, Dickerman A, Dietrich EM, Gabbard JL, Gerdes S, Guard A, Kenyon RW, Machi D, Mao C, Murphy-Olson D, Nguyen M, Nordberg EK, Olsen GJ, Olson RD, Overbeek JC, Overbeek R, Parrello B, Pusch GD, Shukla M, Thomas C, VanOeffelen M, Vonstein V, Warren AS, Xia F, Xie D, Yoo H, Stevens R. 2019. The PATRIC Bioinformatics Resource Center: expanding data and analysis capabilities. Nucleic Acids Res 8:606–612. doi:10.1093/nar/gkz943. PMC7145515 31667520

[113] Edgar RC. 2004. MUSCLE: multiple sequence alignment with high accuracy and high throughput. Nucleic Acids Res 32:1792–1797. doi:10.1093/nar/gkh340. 15034147 PMC390337

[114] Cock PJA, Antao T, Chang JT, Chapman BA, Cox CJ, Dalke A, Friedberg I, Hamelryck T, Kauff F, Wilczynski B, de Hoon MJL. 2009. Biopython: freely available Python tools for computational molecular biology and bioinformatics. Bioinformatics 25:1422–1423. doi:10.1093/bioinformatics/btp163. 19304878 PMC2682512

[115] Stamatakis A. 2014. RAxML version 8: a tool for phylogenetic analysis and post-analysis of large phylogenies. Bioinformatics 30:1312–1313. doi:10.1093/bioinformatics/btu033. 24451623 PMC3998144

[116] Stamatakis A, Hoover P, Rougemont J. 2008. A rapid bootstrap algorithm for the RAxML web servers. Syst Biol 57:758–771. doi:10.1080/10635150802429642. 18853362

[117] Le SQ, Gascuel O. 2008. An improved general amino acid replacement matrix. Mol Biol Evol 25:1307–1320. doi:10.1093/molbev/msn067. 18367465

[118] Letunic I, Bork P. 2021. Interactive Tree Of Life (iTOL) v5: an online tool for phylogenetic tree display and annotation. Nucleic Acids Res 49:293–296. 10.1093/nar/gkab301 PMC8265157 33885785

[119] Page AJ, Cummins CA, Hunt M, Wong VK, Reuter S, Holden MTG, Fookes M, Falush D, Keane JA, Parkhill J. 2015. Roary: rapid large-scale prokaryote pangenome analysis. Bioinformatics 31:3691–3693. doi:10.1093/bioinformatics/btv421. 26198102 PMC4817141

[120] Eren AM, Esen OC, Quince C, Vineis JH, Morrison HG, Sogin ML, Delmont TO. 2015. Anvi’o: an advanced analysis and visualization platform for ‘omics data. PeerJ 3:e1319. doi:10.7717/peerj.1319. 26500826 PMC4614810

