# Supplementary material for: Exploring the Potential Molecular Mechanisms of Interactions between a Probiotic Consortium and Its Coral Host
Source: mSystems. 2023 Jan 23;8(1):e00921-22. doi: 10.1128/msystems.00921-22 (PMC9948713; doi:10.1128/msystems.00921-22)
Supplement: TABLE S1 [file msystems.00921-22-s0001.docx]

**TABLE S1**

| **Treatments** | **Strains** | **BMC-PCR initial screening** |
| --- | --- | --- |
| BMC | BMC1 – *Pseudoalteromonas* sp.  BMC2 - *Pseudoalteromonas* sp.  BMC3 - *Pseudoalteromonas* sp.  BMC4 - *Pseudoalteromonas* sp.  BMC5 - *Pseudoalteromonas* sp.  BMC6 – *Cobetia* sp.  BMC7 – *Halomonas* sp. | Catalase (+++), and *nirK*  Catalase (+), and *nirK*  Catalase (+), *nirK* and *nifH*  Catalase (+++), and *dmdA*  Catalase (+), *nirK, and antagonistic activity*  Catalase (++++)  Catalase (+), and *nifH* |
| Pathogen | *Vibrio coralliilyticus* strain ATCC BAA-450 | - |
| BMC + Pathogen | Both BMC consortium and pathogen | - |
| Control | Saline | - |
