# Supplementary material for: Exploring the Potential Molecular Mechanisms of Interactions between a Probiotic Consortium and Its Coral Host
Source: mSystems. 2023 Jan 23;8(1):e00921-22. doi: 10.1128/msystems.00921-22 (PMC9948713; doi:10.1128/msystems.00921-22)
Supplement: TABLE S4 [file msystems.00921-22-s0004.docx]

**TABLE S4**

|  | **BMC 1**  (GCA_013349945.1) | **BMC 2**  (GCA_013350045.1) | **BMC 3**  (GCA_013349985.1) | **BMC 4**  (GCA_013349995.1) | **BMC 5**  (GCA_013350085.1) | **BMC 6**  (GCA_013350055.1) | **BMC 7**  (GCA_013349955.1) |
| --- | --- | --- | --- | --- | --- | --- | --- |
| **Identification (16s)** | *Pseudoalteromonas* sp | *Pseudoalteromonas* sp | *Pseudoalteromonas* sp | *Pseudoalteromonas* sp | *Pseudoalteromonas* sp | *Cobetia marina* | *Halomonas taeanensis* |
| **Genome integrity** | 87.58% | 100% | 100% | 100% | 100% | 99.14% | 99.89% |
| **Contamination** | 0.19% | 0.66% | 1.02% | 0.68% | 0.43% | 0.43% | 1.74% |
| **Number of base pairs** | 4.213.933 | 4.723.202 | 4.778.378 | 4.940.092 | 4.720.321 | 3.977.722 | 3.836.276 |
| **Number of contigs** | 68 | 116 | 222 | 202 | 109 | 105 | 92 |
| **N50** | 263244 | 356239 | 346736 | 124703 | 356234 | 493696 | 233962 |
| **tRNAs** | 72 | 107 | 108 | 106 | 109 | 72 | 67 |
| **rRNAs** | 6 | 11 | 14 | 13 | 11 | 6 | 3 |
| **Coding genes** | 3868 | 4328 | 4443 | 4656 | 4319 | 3446 | 3575 |
| **Genes encoding hypothetical proteins** | 1189 | 1324 | 1380 | 1649 | 1322 | 852 | 832 |
| **Proportion of proteins linked to known functions** | 69.26% | 69.41% | 68.93% | 64.59% | 69.4% | 75.27% | 76.72% |
| **GC content** | 41.12% | 41.21% | 41.15% | 41.14% | 41.20% | 62.47% | 61.90% |
