# Supplementary material for: Exploring the Potential Molecular Mechanisms of Interactions between a Probiotic Consortium and Its Coral Host
Source: mSystems. 2023 Jan 23;8(1):e00921-22. doi: 10.1128/msystems.00921-22 (PMC9948713; doi:10.1128/msystems.00921-22)
Supplement: TABLE S2 [file msystems.00921-22-s0002.docx]

**TABLE S2**

| **Genome 1** | **Genome 2** | **GGDC** | **fastANI** |
| --- | --- | --- | --- |
|  |  | **DDH estimate %** | **ANI value %** |
| BMC 1, 2, 3, 4 and 5 | *Pseudoalteromonas* sp. CO109Y | 75.50 | 97.20 |
| BMC 1, 2, 3, 4 and 5 | *Pseudoalteromonas shioyasakiensis* JCM 18891 | 74.90 | 97.12 |
| BMC 1, 2, 3, 4 and 5 | *Pseudoalteromonas shioyasakiensis* M1400201 | 70.8 | 96.58 |
| BMC 1, 2, 3, 4 and 5 | *Pseudoalteromonas* sp. P1-8 | 70.60 | 96.56 |
| BMC 1, 2, 3, 4 and 5 | *Pseudoalteromonas shioyasakiensis* SDCH90 | 63.80 | 95.63 |
| BMC 1, 2, 3, 4 and 5 | *Pseudoalteromonas* sp. Bablab jr010 | 52.60 | 93.86 |
| BMC 1, 2, 3, 4 and 5 | *Pseudoalteromonas* sp. Bablab jr011 | 52.30 | 84.12 |
| BMC 1, 2, 3, 4 and 5 | *Pseudoalteromonas lipolytica* UCD-48B | 52.10 | 93.88 |
| BMC 1, 2, 3, 4 and 5 | *Pseudoalteromonas* sp*.* NH153 | 52 | 93.82 |
| BMC 1, 2, 3, 4 and 5 | *Pseudoalteromonas* sp. PA2MD11 | 52 | 93.78 |
| BMC 1, 2, 3, 4 and 5 | *Pseudoalteromonas profundi* MNAD1.6 | 50.90 | 93.50 |
| BMC 1, 2, 3, 4 and 5 | *Pseudoalteromonas shioyasakiensis* D1497 | 44 | 91.72 |
| BMC 1, 2, 3, 4 and 5 | *Pseudoalteromonas* sp. Bablab jr004 | 35.30 | 89.91 |
| BMC 1, 2, 3, 4 and 5 | *Pseudoalteromonas* sp. CO133X | 29.10 | 87.13 |
| BMC 1, 2, 3, 4 and 5 | *Pseudoalteromonas arabiensis* JCM 17292 | 28.30 | 86.47 |
| BMC 1, 2, 3, 4 and 5 | *Pseudoalteromonas donghaensis* HJ51 | 25.40 | 84.32 |
| BMC 1, 2, 3, 4 and 5 | *Pseudoalteromonas lipolytica* CSB02KR | 24.20 | 83.60 |
| BMC 1, 2, 3, 4 and 5 | BMC 1, 2, 3, 4 and 5 | 100 | 97.28–99.99 |
| BMC 6 | *Cobetia amphilecti* N80 | 64.10 | 96.13 |
| BMC 6 | *Cobetia* sp. UBA11601 | 62.70 | 95.83 |
| BMC 6 | *Cobetia* sp. SP288 | 62.10 | 96 |
| BMC 6 | *Cobetia* sp. 4B | 61.90 | 95.89 |
| BMC 6 | *Cobetia* sp. MC34 | 61.90 | 95.89 |
| BMC 6 | *Cobetia* sp. UBA4515 | 61.80 | 95.86 |
| BMC 6 | *Cobetia* sp. SAT113 | 61.70 | 95.88 |
| BMC 6 | *Cobetia marina* KMM296 | 60.90 | 95.74 |
| BMC 6 | *Cobetia amphilecti* B2M13 | 60.80 | 95.69 |
| BMC 6 | *Cobetia* sp. UCD24C | 60.50 | 95.60 |
| BMC 7 | *Halomonas taeanensis* USBA-857 | 41.60 | 91.21 |
| BMC 7 | *Halomonas taeanensis* BH539 | 40.80 | 91.31 |
| BMC 7 | *Halomonas* sp. YLGW01 | 29 | 85.86 |
