# Supplementary figures and images for: Exploring the Potential Molecular Mechanisms of Interactions between a Probiotic Consortium and Its Coral Host
Source: mSystems. 2023 Jan 23;8(1):e00921-22. doi: 10.1128/msystems.00921-22 (PMC9948713; doi:10.1128/msystems.00921-22)

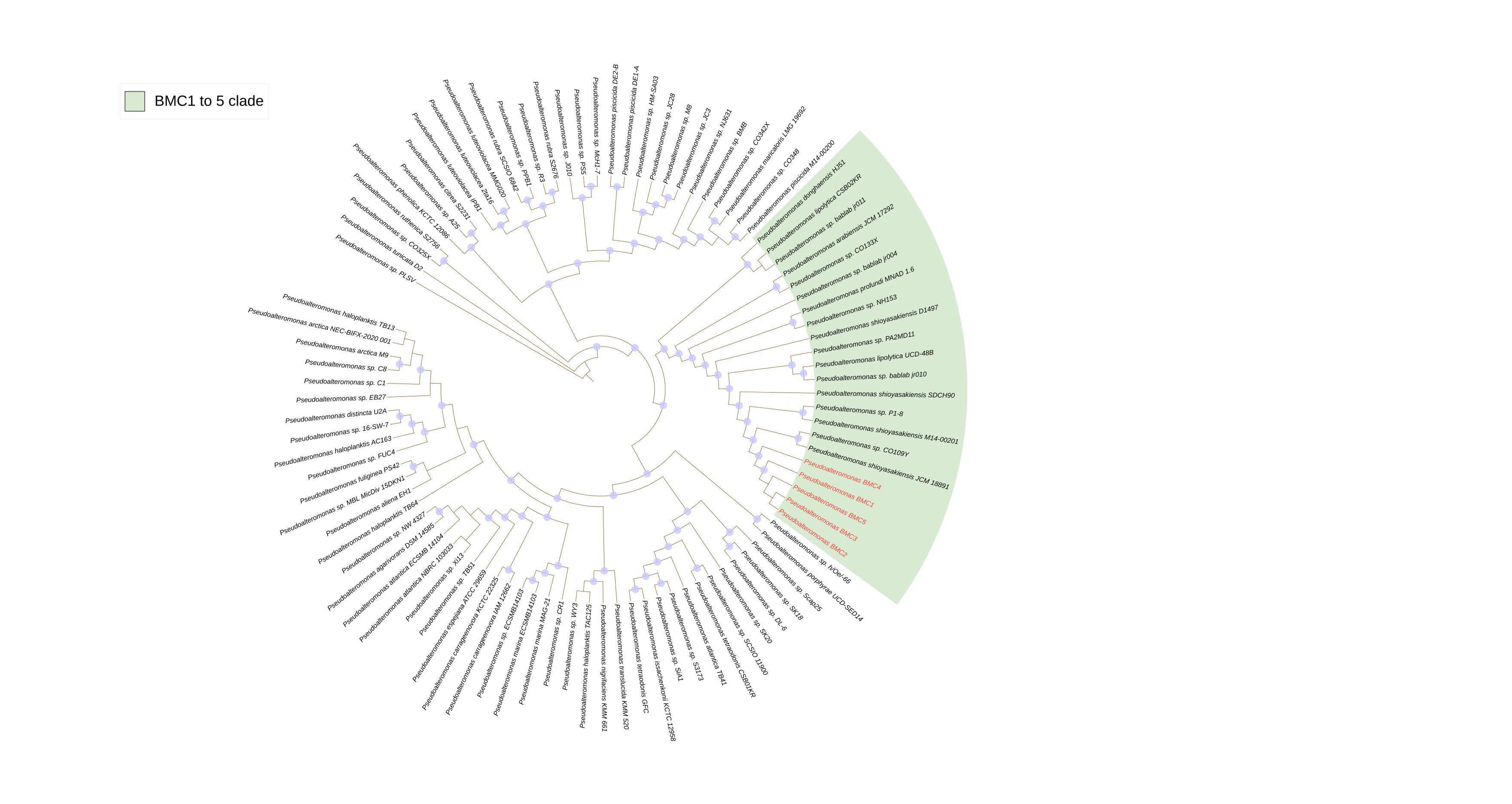

Supplement: FIG S1 [file msystems.00921-22-s0007.tif]
