# Supplementary material for: Exploring the Potential Molecular Mechanisms of Interactions between a Probiotic Consortium and Its Coral Host
Source: mSystems. 2023 Jan 23;8(1):e00921-22. doi: 10.1128/msystems.00921-22 (PMC9948713; doi:10.1128/msystems.00921-22)
Supplement: TABLE S5 [file msystems.00921-22-s0005.docx]

**TABLE S5**

| **Proteins** | **Subsystem** | **Function** | **BMC** |
| --- | --- | --- | --- |
| Superoxide dismutase [Fe] (EC 1.15.1.1) | Oxidative stress | Inactivates superoxide anion radicals that are normally produced within cells | 1 to 7 |
| Glutathione synthetase (EC 6.3.2.3) | Oxidative stress | Synthesizes glutathione from L-cysteine and L-glutamate | 1 to 7 |
| Catalase-peroxidase KatG (EC 1.11.1.21) | Oxidative stress | Bifunctional enzyme with catalase and peroxidase activity | 1 to 7 |
| Hemerythrin domain protein | Oxidative stress | Responsible for transporting oxygen and can act as an oxygen scavenger | 6 |
| Adenosylcobinamide kinase (EC 2.7.1.156) | Cobalamin synthesis | Involved in adenosylcobalamin biosynthesis, which is part of cofactor biosynthesis | 1 to 5 and 7 |
| Adenosylcobinamide-phosphate synthase (EC 6.3.1.10) | Cobalamin synthesis | Part of the cobalamin biosynthetic pathway | 1 to 5 and 7 |
| Betaine Aldehyde Dehydrogenase (EC 1.2.1.8) | Betaine glycine synthesis | Part of the glycine betaine biosynthetic pathway | 1 to 7 |
| Choline dehydrogenase (EC 1.1.99.1) | Betaine glycine synthesis | Part of the betaine biosynthesis pathway from choline | 7 |
| Ectoine hydroxylase | Ectoines synthesis | Involved in the 5-hydroxyectoine biosynthesis | 6 and 7 |
| L-ectoine synthase (EC 4.2.1.108) | Ectoines synthesis | Part of ectoine biosynthesis | 6 and 7 |
| Aerobactin synthase (EC 6.3.2.39) | Siderophores synthesis | Involved in the aerobactin biosynthesis pathway, which is part of siderophore biosynthesis | 7 |
| L-2,4-diaminobutyrate decarboxylase | Siderophores synthesis | Siderophore biosynthesis | 6 |
| Nitrite reductase [NAD (P) H] | Nitrogen cycle | Nitrite reduction | 6 and 7 |
| CoA-transferase / lyase DddD | DMSP degradation | Dimethylsulfide (DMS) producer from DMSP degradation | 6 |
