# Supplementary material for: Exploring the Potential Molecular Mechanisms of Interactions between a Probiotic Consortium and Its Coral Host
Source: mSystems. 2023 Jan 23;8(1):e00921-22. doi: 10.1128/msystems.00921-22 (PMC9948713; doi:10.1128/msystems.00921-22)
Supplement: TABLE S6 [file msystems.00921-22-s0006.docx]

**Table S6**

| **Protein** | **Function** | **BMC** |
| --- | --- | --- |
| Luminescence regulatory protein LuxO | Siderophore production | 1–5 |
| Ferri-bacillibactin esterase BesA | Hydrolysis of ferri-bacillibactin during the trilactone cycle, leading to the cytosolic iron release | 1–5 and 6 |
| Cyclic pyranopterin monophosphate synthase | Molybdopterin biosynthesis. Molybdopterin is a cofactor to xanthine oxidase, DMSO reductase, sulfite oxidase, and nitrate reductase | 6 |
| Heme A synthase | Found in many biologically important hemoproteins such as catalases and heme peroxidase | 6 |
| Plipastatin synthase subunit C | Involved in lipopeptide antibiotic biosynthesis | 6 |
| Nitrite reductase | Involved in the nitrogen cycle | 6 and 7 |
| Siroheme synthase | Involved in sulfur and nitrogen metabolism | 6 and 7 |
| Cobyric acid synthase | Involved in the synthesis of adenosylcobalamin (B12) | 7 |
| Cobalamin biosynthesis protein CobD | Involved in the synthesis of adenosylcobalamin (B12) | 7 |
| Aerobactin synthase | Siderophore production | 7 |
| Bifunctional adenosylcobalamin biosynthesis protein CobP | Involved in the synthesis of adenosylcobalamin (B12) | 7 |
| Catalase-peroxidase 1 (KatG1) | Involved in protecting against oxidative stress | 7 |
| Dihydrofolate synthase / folylpolyglutamate synthase | Involved in the synthesis of folate (B9) | 7 |
| Dimethlysulfonioproprionate lyase DddP | Involved in the DMSP degradation pathway | 7 |
| Flavohemoprotein | Involved in protecting against nitrosative stress | 7 |
| Hydrogen peroxide-inducible genes activator | Involved in protecting against oxidative stress | 7 |
| L-lysine N6-monooxygenase | Siderophore production | 7 |
| N(2)-citryl-N(6)-acetyl-N(6)-hydroxylysine synthase | Siderophore production | 7 |
| Nitrate reductase | Involved in the nitrogen cycle | 7 |
| Thiamine-monophosphate kinase | Involved in the synthesis of thiamine-pyrophosphate (B1) | 7 |
| Urease subunit beta | Involved in the nitrogen cycle | 7 |
| Ubiquinone biosynthesis O-methyltransferase | Involved in protecting against oxidative stress | 7 |
| Pantothenate synthetase | Involved in the synthesis of pantothenate (B5) | 7 |
