# Supplementary material for: Exploring the Potential Molecular Mechanisms of Interactions between a Probiotic Consortium and Its Coral Host
Source: mSystems. 2023 Jan 23;8(1):e00921-22. doi: 10.1128/msystems.00921-22 (PMC9948713; doi:10.1128/msystems.00921-22)
Supplement: TABLE S3 [file msystems.00921-22-s0003.docx]

**TABLE S3**

| **Genome** | **BioProject Accession** | **BioSample Accession** |
| --- | --- | --- |
| *Pseudoalteromonas lipolytica* CSB02KR | PRJNA328511 | SAMN05374560 |
| *Pseudoalteromonas shioyasakiensis* SDCH90 | PRJNA737135 | SAMN19682664 |
| *Pseudoalteromonas* sp. PA2MD11 | PRJNA674396 | SAMN16655686 |
| *Pseudoalteromonas* sp. bablab_jr011 | PRJNA646503 | SAMN15545061 |
| *Pseudoalteromonas arabiensis* JCM 17292 | PRJNA309914 | SAMN04442128 |
| *Pseudoalteromonas donghaensis* HJ51 | PRJNA487231 | SAMN09939547 |
| *Pseudoalteromonas shioyasakiensis* D1497 | PRJNA643440 | SAMN15415016 |
| *Pseudoalteromonas shioyasakiensis* JCM 18891 | PRJNA309917 | SAMN04442129 |
| *Pseudoalteromonas* sp. NH153 | PRJNA309918 | SAMN04442130 |
| *Pseudoalteromonas* sp. bablab_jr004 | PRJNA646503 | SAMN15545054 |
| *Pseudoalteromonas* sp. bablab_jr010 | PRJNA646503 | SAMN15545060 |
| *Pseudoalteromonas profundi* MNAD 1.6 | PRJNA491354 | SAMN10075489 |
| *Pseudoalteromonas* sp. CO109Y | PRJNA521722 | SAMN10909769 |
| *Pseudoalteromonas* sp. CO133X | PRJNA521722 | SAMN10909770 |
| *Pseudoalteromonas* sp. P1-8 | PRJNA294442 | SAMN04026652 |
| *Pseudoalteromonas lipolytica* UCD-48B | PRJNA295758 | SAMN04088200 |
| *Pseudoalteromonas shioyasakiensis* M14-00201 | PRJNA795272 | SAMN24777657 |
| *Cobetia amphilecti* B2M13 | PRJNA478695 | SAMN19350924 |
| *Cobetia* sp. UCD-24C | PRJNA295759 | SAMN04088201 |
| *Cobetia* sp. 4B | PRJNA648281 | SAMN15637445 |
| *Cobetia marina* KMM 296 | PRJNA259325 | SAMN03003781 |
| *Cobetia* sp. MC34 | PRJNA678481 | SAMN16632794 |
| *Cobetia amphilecti* N-80 | PRJNA765725 | SAMN21583668 |
| *Cobetia* sp. SP288 | PRJNA391943 | SAMN07618555 |
| *Cobetia* sp. SAT113 | PRJNA391943 | SAMN07618554 |
| *Cobetia* sp. UBA4515 | PRJNA348753 | SAMN06450766 |
| *Cobetia* sp. UBA11601 | PRJNA417962 | SAMN08019747 |
| *Halomonas* sp. YLGW01 | PRJNA664300 | SAMN16205224 |
| *Halomonas taeanensis* USBA-857 | PRJNA330502 | SAMN05421792 |
| *Halomonas taeanensis* BH539 | PRJEB15885 | SAMN05216571 |
